# Uncovering the Underlying Mechanisms of Cancer Metabolism through the Landscapes and Probability Flux Quantifications

**DOI:** 10.1016/j.isci.2020.101002

**Published:** 2020-03-25

**Authors:** Wenbo Li, Jin Wang

**Affiliations:** 1 State Key Laboratory of Electroanalytical Chemistry, Changchun Institute of Applied Chemistry, Chinese Academy of Sciences, Changchun, Jilin 130022, China; 2 Department of Chemistry and Physics, State University of New York at Stony Brook, Stony Brook, NY 11794-3400, USA

**Keywords:** Gene Network, Mathematical Biosciences, Cancer, Metabolic Flux Analysis

## Abstract

Cancer metabolism is critical for understanding the mechanism of tumorigenesis, yet the understanding is still challenging. We studied gene-metabolism regulatory interactions and quantified the global driving forces for cancer-metabolism dynamics as the underlying landscape and probability flux. We uncovered four steady-state attractors: a normal state attractor, a cancer OXPHOS state attractor, a cancer glycolysis state attractor, and an intermediate cancer state attractor. We identified the key regulatory interactions through global sensitivity analysis based on the landscape topography. Different landscape topographies of glycolysis switch between normal cells and cancer cells were identified. We uncovered that the normal state to cancer state transformation is associated with the peaks of the probability flux and the thermodynamic dissipation, giving dynamical and thermodynamic origin of cancer formation. We found that cancer metabolism oscillations consume more energy to support cancer malignancy. This study provides a quantitative understanding of cancer metabolism and suggests a metabolic therapeutic strategy.

## Highlights

• We uncovered cancer network involving gene regulations and metabolic pathways
• We uncovered the emergence of normal state and three cancer metabolic states
• Flux and entropy production rate provide predictors for emergence of cancer states
• This study provides a metabolic therapeutic strategy based on landscape-flux theory

## Graphical Abstract

**Figure:**
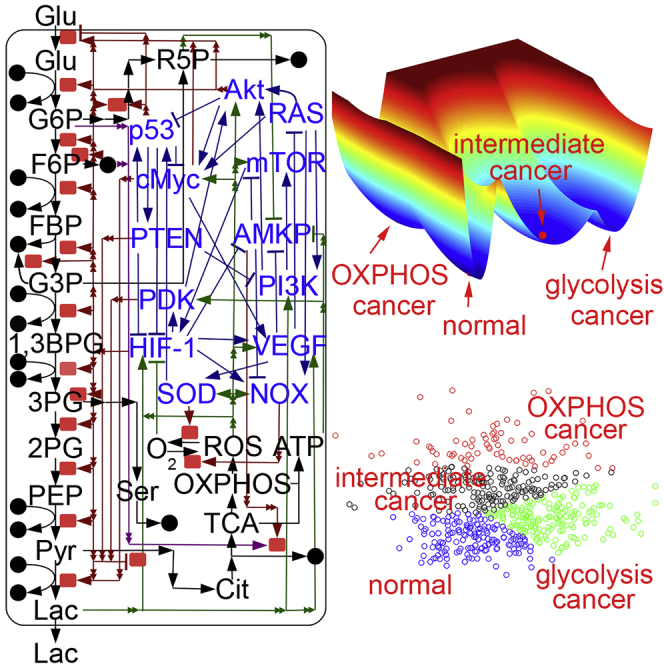

## Introduction

Cancer cells acquire specific biological capabilities to sustain self-replication and survival during tumorigenesis and development. One of the most important emerging hallmarks of cancer is reprogramming of energy metabolism (Hanahan and Weinberg, 2011). For generating new biomass and energy needed in the rapid cell cycle, cancer cells struggle to acquire necessary nutrients from a frequently nutrient-poor environment in varied ways (Pavlova and Thompson, 2016). Two major metabolic pathways, glycolysis and oxidative phosphorylation (OXPHOS), are utilized for producing energies in cancer cells. Most types of cancer cells are observed to have high uptake of glucose but divert glucose-derived pyruvate away from the mitochondrial TCA cycle to lactate production. This is known as “aerobic glycolysis” or the Warburg effect (Warburg, 1956). Aerobic glycolysis not only is important for energy production, but also profits the biosynthesis and rapid cell proliferation (Heiden et al., 2009). In contrast, observations also showed that cancer cells can utilize OXPHOS for ATP production from glucose, fatty acids, or glutamine oxidation (Lehuédé et al., 2016). Although significant progresses have been made, the understanding of the underlying mechanism and the interplay between the two pathways of cancer metabolism is still challenging.

To address these issues, one needs to explore the coupling between the underlying gene regulatory network and metabolic pathway for determining the cancer metabolism. Mathematic models are useful and effective for describing gene networks and metabolic pathways. However, very limited models on cancer metabolism have been suggested. Cancer glycolysis rate was studied based on enzyme kinetics and experimental data from rodent AS-30D hepatoma and human cervix HeLa cells (Marín-Hernández et al., 2011). In the further research (Marín-Hernández et al., 2014), the role of differential expressions of glycolytic enzyme isoforms under different glucose levels was investigated. Roy and Finley (2017) built a model of pancreatic cancer combining metabolic pathways and cell growth. This cancer model involved both metabolites and gene regulations for studying the relationship among HIF-1, AMPK, and ROS (Yu et al., 2017), but involved only several gene nodes without metabolic reactions. Further improved model coupled the gene regulations with metabolic pathways. However, only limited genes and metabolites were included for the study (Jia et al., 2019). It has been reported that not only the enzymes expressed by genes can influence metabolic reaction rates but also the alteration of metabolite level in cancer cell can influence the gene regulations (Pavlova and Thompson, 2016). Thus, a more comprehensive model including both gene regulatory network and metabolic pathways in detail for cancer metabolism from an integrative biological networks perspective is in demand.

Global quantification of the network dynamics is important for understanding the biological process, function, and the underlying mechanism. This can be realized through the identification of the driving forces for the dynamics as the landscape and the probability flux (Wang et al., 2008, Wang et al., 2011, Wang, 2015). The landscape and probability flux has been shown to drive the dynamics of various cancer gene regulatory networks (Li and Wang, 2014, Li and Wang, 2015, Chen and Wang, 2016, Yu and Wang, 2016), giving rise to a global and physical description. However, the underlying mechanism for cancer metabolism still remains elusive.

In this study, we develop an integrative network model including cancer-related metabolic pathway and gene regulatory network. The network includes 13 genes, 17 enzymes, and 23 metabolites, with a total of 53 nodes. The network includes gene-gene regulations, gene-enzyme regulations, and enzyme-catalyzed reactions. We have quantitatively investigated the integrative gene-metabolic network. We uncovered the underlying landscape of cancer metabolism. Four steady-state attractors, normal state, cancer OXPHOS state, cancer glycolysis state, and cancer intermediate sate, emerge based on the landscape topography. Through global sensitivity analysis of the underlying landscape topography, we identified the key gene-gene regulations for promoting cancer OXPHOS state and cancer glycolysis state. Moreover, we observed that normal state to cancer state transformation or the bifurcations are associated with the peaks of the probability flux and the associated entropy production rate. This provides a physical origin and a quantitative indicator of the cancer formation. We also uncovered the underlying mechanism of cancer metabolism oscillations. We make predictions on the effectiveness of various metabolic therapeutic targets. This also provides the therapeutic targets for cancer metabolism oscillations.

## Results

### Cancer Gene-Metabolism Integrative Network

Gene regulatory network and metabolic pathway can interact or regulate with each other. Genes are translated to proteins, and these proteins are assembled to form enzymes in cytoplasm. The enzyme levels control metabolic reaction rates directly. On the other hand, metabolites influence not only the enzymes activity but also the gene expressions indirectly (Pavlova and Thompson, 2016).

**Figure 1:**
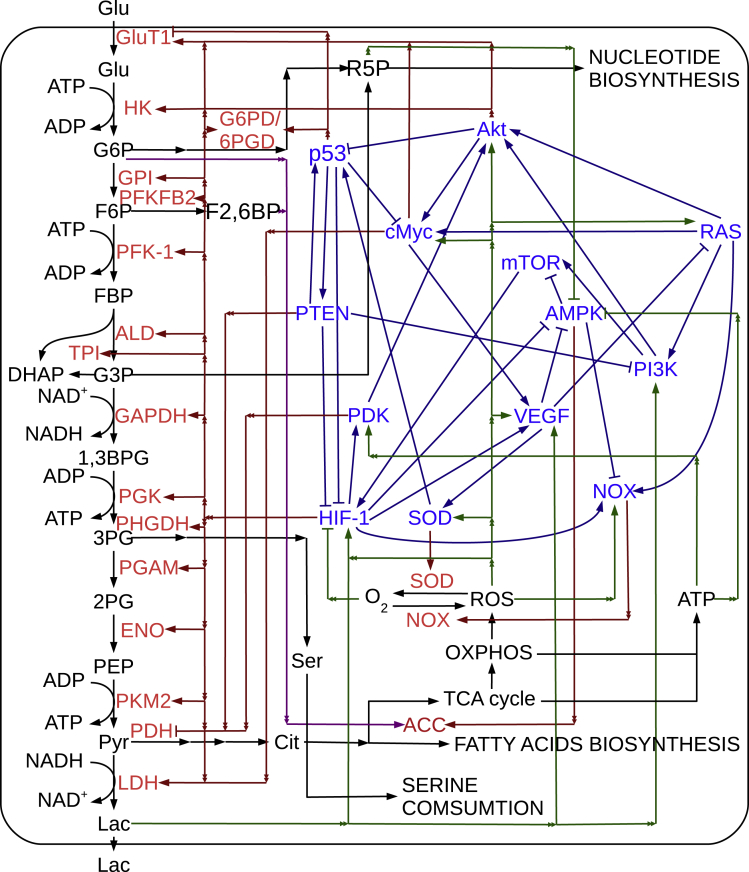
Cancer Gene-Metabolism Integrative Network Genes are colored with blue. Enzymes are colored with red. Metabolites are colored with black. Dark blue arrows and bars represent gene-gene interactions. Dark red arrows and bars represent and gene-enzyme regulations. Purple arrows and bars represent metabolite-enzyme regulations. Black arrows represent biochemical reactions. Double arrows represent shared lines for multiple regulations. Each of the same colored connections that start with double arrows and end with solid arrows and bars represent one regulation. G6P→→→→R5P: G6P→6PGL→6PGC→Ru5P→R5P, 3PG→→→Ser: 3PG→3PHP→P-ser→Ser, Pyr→→Cit: Pyr→Ac-CoA→Cit.

Our cancer gene-metabolism integrative network includes two parts, gene regulatory network and metabolic pathway. The gene regulatory network includes 13 genes, Akt, AMPK, cMyc, HIF-1, mTOR, NOX, p53, PDK, PI3K, PTEN, RAS, SOD, and VEGF. The metabolic switch promoting deregulated growth is often triggered by mutations in signaling pathways that rest at the crux of anabolic and energetic homeostasis, such as HIF-1a, PI3K/AKT, mTOR, and AMPK (Yizhak et al., 2015). Seventy-three gene-gene, gene-enzyme, metabolite-gene, or metabolite-enzyme interactions were included and listed in Tables S1 and S2. All these interactions were selected from the previous experimental studies (Courtnay et al., 2015, Hammad et al., 2016, Hasawi et al., 2014, Justus et al., 2015, Lien et al., 2016, Prasad et al., 2017, Saunier et al., 2015, Wegner et al., 2015) and EVEX database (Landeghem et al., 2013). For metabolic pathways, we focused on glycolysis pathway and TCA cycle from previous studies (Heiden and DeBerardinis, 2017, Yizhak et al., 2015, Pavlova and Thompson, 2016). The pathway includes 23 metabolites and 17 enzymes along with the related reactions listed in Table S3. Extracellular glucose is transported into cell first. Along with the sequence of reactions of glycolysis, one molecule of intracellular glucose is metabolized into two molecules of pyruvate (pyr) and generates two molecules of ATP. Two main further sequence reactions of pyruvate are important. The pyruvate is reduced by NADH to form lactate, and it is expelled out of the cell. Pyruvate also can be converted to acetyl coenzyme A (acetyl CoA) in mitochondria. Acetyl CoA enters into the TCA cycle, and oxidative phosphorylation is in sequence for generating more ATP. The whole network is shown in Figure 1. The sequential metabolic reactions, which are displayed as the sequential arrows, such as Pyr→→Cit, can be treated effectively as one comprehensive reaction including the substrates in the first reaction and the products in the last reaction. The sequential metabolic reactions were modeled as one comprehensive reaction. Thus, the ODEs (Ordinary Differential Equations) exclude these intermediate metabolites for simplicity. For the clarity of the network display, we did not show certain metabolites, which are not included in the ODEs. The genes NOX and SOD are in the gene network. Their translated proteins also control the related metabolic reactions. To avoid the redundancy in the model, the gene and related protein level are represented as one variable in our model.

The gene-gene regulatory network and the metabolic pathway are mainly connected by the gene-enzyme and metabolite-gene interactions that bridge the two networks together. As shown in Figure 1, the metabolic pathways are controlled by Akt, p53, cMyc, PTEN, HIF-1, and PDK genes, whereas the gene regulatory network is influenced by lactate, ROS, ATP, and O_2_. Besides, the metabolic enzymes can be regulated by metabolites, such as G6P and F2,6BP. These genes, enzymes, metabolites, and interactions among them compose a cancer gene-metabolism integrative network.

Owing to the different dynamical characteristics between gene-gene regulatory interactions and metabolic reactions, we consider the driving force for the network dynamics differently. The driving forces of the dynamics for the gene expressions or enzyme levels are determined by:

(Equation 1) $$\dot{X_{i}}=F\left(X_{i}\right)=A_{i}\prod_{j=1}^{N_{i}}H_{ji}-D_{i}X_{i}$$

(Equation 2) $$H_{ji}=\frac{S_{ji}^{n}}{S_{ji}^{n}+X_{j}^{n}}+\gamma_{ji}\frac{X_{j}^{n}}{S_{ji}^{n}+X_{j}^{n}}$$

(Equation 3) $$=\left(\gamma_{ji}-1\right)\frac{X_{j}^{n}}{S_{ji}^{n}+X_{j}^{n}}+1$$

(Equation 4) $$=\left(1-\gamma_{ji}\right)\frac{S_{ji}^{n}}{S_{ji}^{n}+X_{j}^{n}}+\gamma_{ji}$$

Here, *F* represents the driving force of the variable *X*, the level of gene expressions or enzyme. *A* represents the basic production rate of the gene or the enzyme. *D* represents the degradation rate of the gene or the enzyme. *S* represents the gene expression level with half threshold of production. The parameter *n* is the Hill coefficient for describing the cooperativity of the interactions. $H_{ji}$ is described by a nonlinear function, namely, the shifted Hill function (Lu et al., 2013, Lu et al., 2014). The positive parameter $\gamma_{ji}$ represents the activation of $X_{i}$ from $X_{j}$ if γ>1 and the inhibition if γ<1. For the gene-gene regulatory interactions, $H_{ji}$ is the summation of two Hill functions, the inhibition term and the activation term. When γ>1, the Eq. 2 can be converted into Eq. 3, the activation term (only the second term) is effective. Conversely, when γ<1, the Eq. 2 can be converted into Eq. 4, only the inhibition term is effective. The parameters for this cancer metabolism model are chosen carefully for producing the results that are biologically relevant and reasonable. The interactions strengths are listed in Table S4.

The driving forces of the dynamics for the metabolite concentration are determined by:

(Equation 5) $$\dot{Y_{i}}=F\left(Y_{i}\right)=\sum_{j=1}^{N_{i}}X_{j}r_{j}$$

F represents the driving force of the variable Y, the concentration of metabolite. It describes the summation of enzyme kinetic velocity $r_{j}$ multiplied by the related enzymes $X_{j}$. The kinetic equation is from previous studies. Details are in Transparent Methods. The related parameters are listed in Table S5.

In real dynamics, fluctuations are unavoidable. When including these effects, the above deterministic equations become stochastic. One then targets the corresponding probability evolution rather than the trajectory evolution. This is because the trajectory evolution now is stochastic and unpredictable, whereas the probability evolution is deterministic and predictable. Such probabilistic evolution equation is often in the form of the Fokker-Planck diffusion equation in the continuous variable representation. The steady-state probability landscape and the corresponding probability flux can be obtained by either the self-consistent mean field approach or by the Langevin simulations.

### Landscape of Cancer Metabolism

The dynamics for non-equilibrium system (here, the gene-metabolic network dynamics) are determined by two driving forces: the underlying landscape and the probability flux (Wang, 2015). The landscape reflects the steady-state probability or weight of the corresponding state. Functional states with higher chances of being observed can be quantified by the basin of attractions on the landscape. The stability of the function can thus be determined by the barrier height or the time escaping from the basin of attraction. Therefore, this can provide a global characterization and a stability measure in terms of the landscape topography. The landscape has the tendency to attract the system down to the gradient. The probability flux refers to the steady-state probability flux. It reflects the tendency for leading the system dynamics to rotate around. It measures the degree of how far away from the equilibrium is. The dynamics of the underlying gene-metabolic networks can then be viewed as a charged particle moving in an electric field (landscape) and magnetic field (probability flux). Details are in Transparent Methods.

The landscape of cancer metabolism can be quantified based on the integrative network of cancer gene regulatory-metabolic pathway, using the self-consistent mean field approximation of the corresponding probabilistic evolution equation or by direct stochastic simulations. The landscape *U* is defined as $U=-ln\left(P_{ss}\right)$, which is directly related to steady-state probability distribution $P_{ss}$ of the concentration or expression variables (see Transparent Methods).

**Figure 2:**
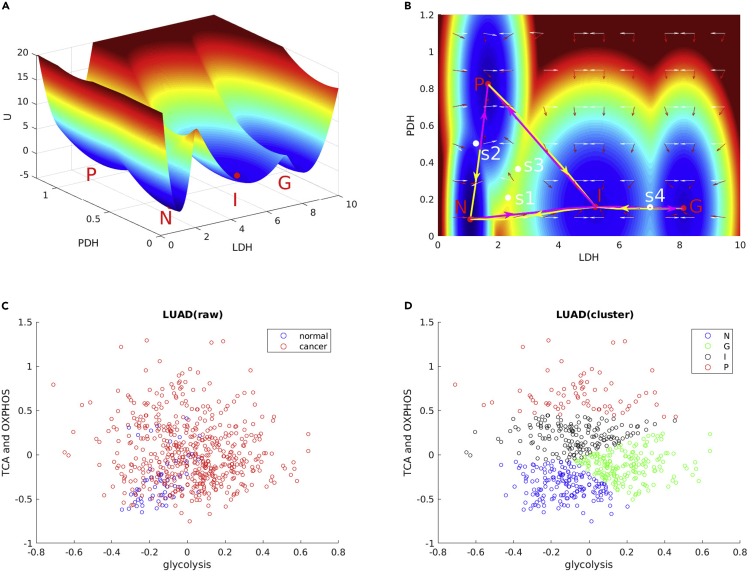
Landscape of Cancer Gene-Metabolism and Related Gene Expressions from GDC The landscape is represented in terms of LDH expression level and PDH expression level. N, normal state; P, cancer OXPHOS state; G, cancer glycolysis state; I, cancer intermediate state. s1, saddle between normal state and cancer intermediate state; s2, saddle between normal state and cancer OXPHOS state; s3, saddle between cancer intermediate state and cancer OXPHOS state; s4, saddle between cancer intermediate state and cancer glycolysis state. The yellow arrows represent the paths from N to I, from I to P, from N to P, and from I to G; the magenta arrows represent the paths from I to N, from P to N, from P to I, and from G to I. The white arrows represent the directions of the steady-state probability flux, and the red arrows represent the directions of the negative gradient of the potential landscape. (A) The landscape of cancer gene-metabolism in 3D. (B) The landscape of cancer gene-metabolism in 2D. (C) Gene expression data with normal and cancer samples. (D) Gene expression data clustered by K-means.

The dynamical equations of cancer gene-metabolism integrative network includes 53 variables involving genes, enzymes and metabolites. It is difficult to visualize the landscape in 53 dimensions. Therefore, we choose two dimensions for display by integrating out other dimensions. Lactate dehydrogenase (LDH) is a key enzyme for switching away from TCA cycle and can reflect aerobic glycolysis flux. Pyruvate dehydrogenase (PDH) is the first enzyme component of the pyruvate dehydrogenase complex (PDC), which contributes to transforming pyruvate into mitochondria for subsequential TCA cycle and oxidative phosphorylation. In order to describe the different characteristics of the cancer cells in generating the energies, we choose LDH and PDH as the two-dimensional variables. Four steady-state attractors, normal state (N), cancer OXPHOS state (P), cancer glycolysis state (G), and cancer intermediate state (I) attractors, emerge and are shown in Figures 2A and 2B. It is obvious that the LDH/PDH level of the cancer intermediate state is lower compared with either the cancer OXPHOS state or the cancer glycolysis state. The red region represents high potential area, whereas the blue region represents the low potential area. Between the two steady-state attractors, there is a saddle, which is colored white in Figure 2B. We define the saddle between normal state and cancer intermediate state as s1, the saddle between normal state and cancer OXPHOS state as s2, the saddle between cancer intermediate state and cancer OXPHOS state as s3, and the saddle between cancer intermediate state and cancer glycolysis state as s4.

Cancer cells display distinct metabolic features with different tissues (Lehuédé et al., 2016). As shown in Figures 2A and 2B, normal state does not need big ATP consumptions compared with the cancer cells. The levels of LDH and PDH are low. The level of PDH for the cancer OXPHOS state is much higher than that of the normal state. This corresponds mainly to the oxidative phosphorylation for ATP generation, related to the oxidative-phosphorylation-dependent cancer type such as melanomas and glioblastomas (Obre and Rossignol, 2015). On the other hand, the level of LDH for the cancer glycolysis state is much higher than that of the normal state. This corresponds mainly to the glycolysis for ATP generation, related to glycolysis-dependent cancer type such as liver and colorectal cancers (Ma et al., 2013, Amann and Hellerbrand, 2009, Calvisi et al., 2011, Haber et al., 1998, Graziano et al., 2016). The cancer intermediate state has less PDH level compared with the cancer OXPHOS state and less LDH level compared with the cancer glycolysis state. This may correspond to the mixed cancer phenotype such as prostate cancer (Elia et al., 2015). The cancer intermediate state also bridges the normal state, the cancer OXPHOS state, and the cancer glycolysis state. The normal, OXPHOS, and glycolysis states can switch to each other through the cancer intermediate state.

The above results predicted from our theoretical models have been observed in the experiments in several cancer types. As an example, we utilize the RNA sequencing (RNA-seq) data of lung adenocarcinoma (LUAD) from Genomic Data Commons Data Portal (GDC). The dataset of lung adenocarcinoma reveals three disease types: acinar cell neoplasms; adenomas and adenocarcinomas; and cystic, mucinous, and serous neoplasms. It includes more disease types than most of other datasets from GDC and has many normal samples. The complexity of this dataset can ensure that all the major cancer states (glycolysis cancer state, OXHPHOS cancer state, and intermediate cancer state) can be displayed from RNA-seq data. The gene expressions related to glycolysis, TCA cycle, and OXPHOS are selected for further analysis. Since it is hard to visualize multiple genes, we put these genes into two groups. One group contains glycolysis-related genes, and the other group contains TCA cycle- and OXPHOS-related genes. These related genes in groups are listed in Table S6. Then we normalize every gene expression and average the gene expressions in each group, respectively. The two mean gene expressions are used for describing the level of glycolysis and OXPHOS, respectively, as shown in Figures 2C and 2D. In Figure 2C, it is obvious that the glycolysis and OXPHOS levels of normal cells are much lower than that of cancer cells. This corresponds to the normal state (N) along with the cancer states (G,I,P) in the results of our model. We further cluster these expressions into four groups as shown in Figure 2D. The four groups are consistent with the four states, normal state, cancer glycolysis state, cancer OXPHOS state, and cancer intermediate state, from the results of our model. These trends and results have also been observed in other cancer types, lung squamous cell carcinoma (LUSC), cervical squamous cell carcinoma and endocervical adenocarcinoma (CESC), and uterine corpus endometrial carcinoma (UCEC), as shown in Figure S1.

To further quantify the possible switching processes among steady-state attractors, we identified the dominant paths between different attractors by minimizing the transition actions. The dominant paths are shown on the landscape in Figure 2B. The yellow arrows (from N state to I state, from I state to P state, from N state to P state, and from I state to G state) represent the tumorigenesis of OXPHOS or glycolysis cancer type, whereas the magenta arrows (from I state to N state, from P state to N state, from P state to I state, and from G state to I state) represent the cancer recovery or switching to the mixed cancer type. We also show the steady-state probability flux of the cancer metabolism landscape in Figure 2B. The white and red arrows, respectively, represent the direction of the steady-state probability flux and the negative gradient of the potential landscape. The dynamics of the cancer metabolic network is determined by both the gradient of the potential and the steady-state probability flux. The force from the steady-state probability flux leads to the dominant paths to deviate from the conventionally expected potential gradient paths. As we can see the forward and back dominant paths between the normal state and any cancer state are different from each other to different extents. In other words, the dominant paths for cancer tumorigenesis and caner recovery are not necessarily reversible. Furthermore, the switchings between the cancer types are also not necessarily reversible.

### Global Sensitivity Analysis of the Cancer Metabolism Based on Landscape Topography

**Figure 3:**
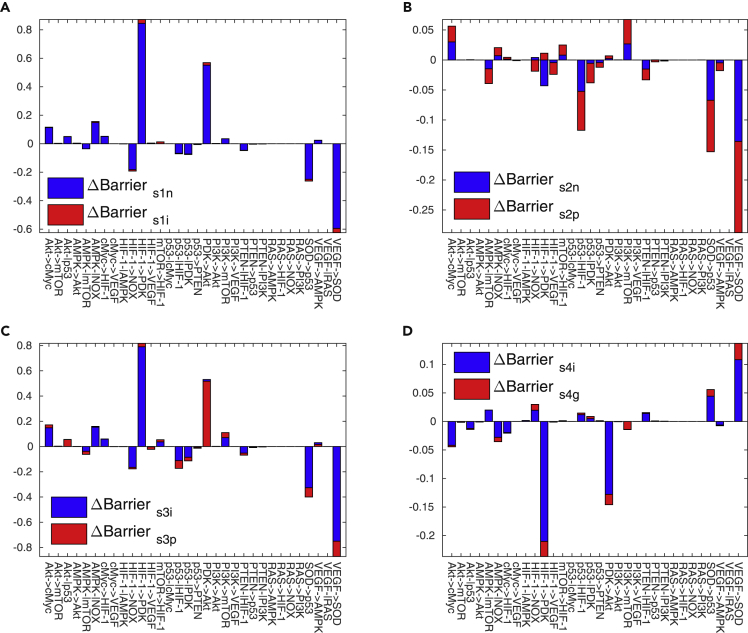
Global Sensitivity Analysis for the 32 Gene-Gene Regulations (A) The changes of the barriers from s1 to normal steady state and cancer intermediate state. (B) The changes of the barriers from s2 to normal steady state and cancer OXPHOS state. (C) The changes of the barriers from s3 to cancer intermediate state and cancer OXPHOS state. (D) The changes of the barriers from s3 to cancer intermediate state and cancer glycolysis state. X axis represents the 32 gene-gene regulations. Y axis represents the barrier changes. Each parameter is increased by 1% individually. ΔBarrier_*s*1*n*_: the change of the barrier from s1 to normal steady state. ΔBarrier_*s*1*i*_: the change of the barrier from s1 to cancer intermediate state. ΔBarrier_*s*2*n*_: the change of the barrier from s2 to normal state. ΔBarrier_*s*2*p*_: the change of the barrier from s2 to cancer OXPHOS state. ΔBarrier_*s*3*n*_: the change of the barrier from s3 to normal state. ΔBarrier_*s*3*i*_: the change of the barrier from s3 to cancer intermediate state. ΔBarrier_*s*4*i*_: the change of the barrier from s4 to cancer intermediate state. ΔBarrier_*s*4*g*_: the change of the barrier from s4 to cancer glycolysis state.

We define the potential difference from the saddle to the steady-state attractor as the barrier height. It represents the ability of switching from one steady-state attractor to the another. According to Figures 2A and 2B, we can quantify the barrier from s1 to normal steady state (Barrier_*s*1*n*_) and the barrier from s1 to cancer OXPHOS steady state (Barrier_*s*1*p*_). Similarly, we can quantify the barrier from s2 to normal steady state (Barrier_*s*2*n*_), the barrier from s2 to cancer intermediate steady state (Barrier_*s*2*i*_), the barrier from s3 to cancer intermediate steady state (Barrier_*s*3*i*_) and the barrier from s3 to cancer OXPHOS steady state (Barrier_*s*3*p*_), and the barrier from s4 to cancer intermediate steady state (Barrier_*s*4*i*_) and the barrier from s4 to cancer glycolysis steady state (Barrier_*s*4*g*_). Each of the 73 gene-gene, gene-enzyme, and metabolite-gene interaction parameters is increased by certain percentages for perturbing the network, leading to the changes in the respective barrier as shown in Figures 3, S2, and S3.

Interestingly, the barrier changes from s4 to cancer OXPHOS state and from s4 to glycolysis cancer state are opposite, shown in Figures 3C and 3D, illustrating the competing nature of the two metabolic pathways of cancer. It also implies that the formation of different cancer type is caused by distinct gene-gene regulations. The global sensitivity analysis based on the landscape topography via barrier heights predicts certain key gene-gene regulations, including Akt->cMyc, Akt-|p53, AMPK-|mTOR, AMPK-|NOX, cMyc->HIF-1, HIF-1>NOX, HIF-1->PDK, p53->HIF-1, P53->PDK, PDK->Akt, PI3K->mTOR, PTEN-|HIF-1, SOD->P53, and VEGF->SOD. HIF-1 and p53 emerge frequently in these predicted important gene-gene regulations. It has been reported that p53 responds to metabolic changes and influences the metabolic pathways through several mechanisms (Vousden and Ryan, 2009). HIF-1 plays an important role in activating transcription of genes encoding glucose transporters and glycolytic enzymes (Semenza, 2010).

We consider that the regulations for promoting cancer OXPHOS state if the barrier changes of the cancer OXPHOS state are more than that of the cancer intermediate state. This is because the cancer OXPHOS state becomes more stable compared with the intermediate state. In a similar way, the regulation changes are for promoting the cancer glycolysis state if the barrier changes of the cancer state are more than that of the intermediate state. For these gene-gene regulations, the most important ones for promoting cancer OXPHOS state are PI3K->mTOR, HIF-1->PDK, Akt->cMyc, mTOR->HIF-1, AMPK-|NOX, cMyc->HIF-1, HIF-1-|AMPK, and Akt->mTOR, whereas the most important ones for promoting the cancer glycolysis state are VEGF->SOD, SOD->p53, HIF-1->NOX, p53-|PDK, p53-|HIF-1, PTEN-|HIF-1, p53->PTEN, mTOR->HIF-1, PTEN->p53, AMPK-|mTOR, and HIF-1-|AMPK. Akt->cMyc is a major signaling pathway for survival in the lymphoid cell (Domínguez-Cáceres et al., 2004). PDK is upregulated by HIF-1 in the lymphocyte cell line. Activation of the PI3K/mTOR signaling pathway is recurrent in different lymphoma types (Tarantelli et al., 2017). Lymphomas belong to the OXPHOS-dependent cancer type (Lehuédé et al., 2016). Activation of the PI3K/Akt/mTOR pathway caused by aberrations at numerous points of genes contributes to the development of breast cancer (McAuliffe et al., 2010). The regulation mTOR->HIF-1 enhanced the expression of GLUT1, which is an important enzyme for glycolysis (Lien et al., 2016). Increasing of NOX could be caused by promoting of HIF-1->NOX. NOX has also been identified as a major source of ROS in endothelial cells (Diebold et al., 2012). It has been reported that an increase of ROS profits glycolysis (Molavian et al., 2016).

### Landscape Topography Changes upon Changes in Important Regulations

**Figure 4:**
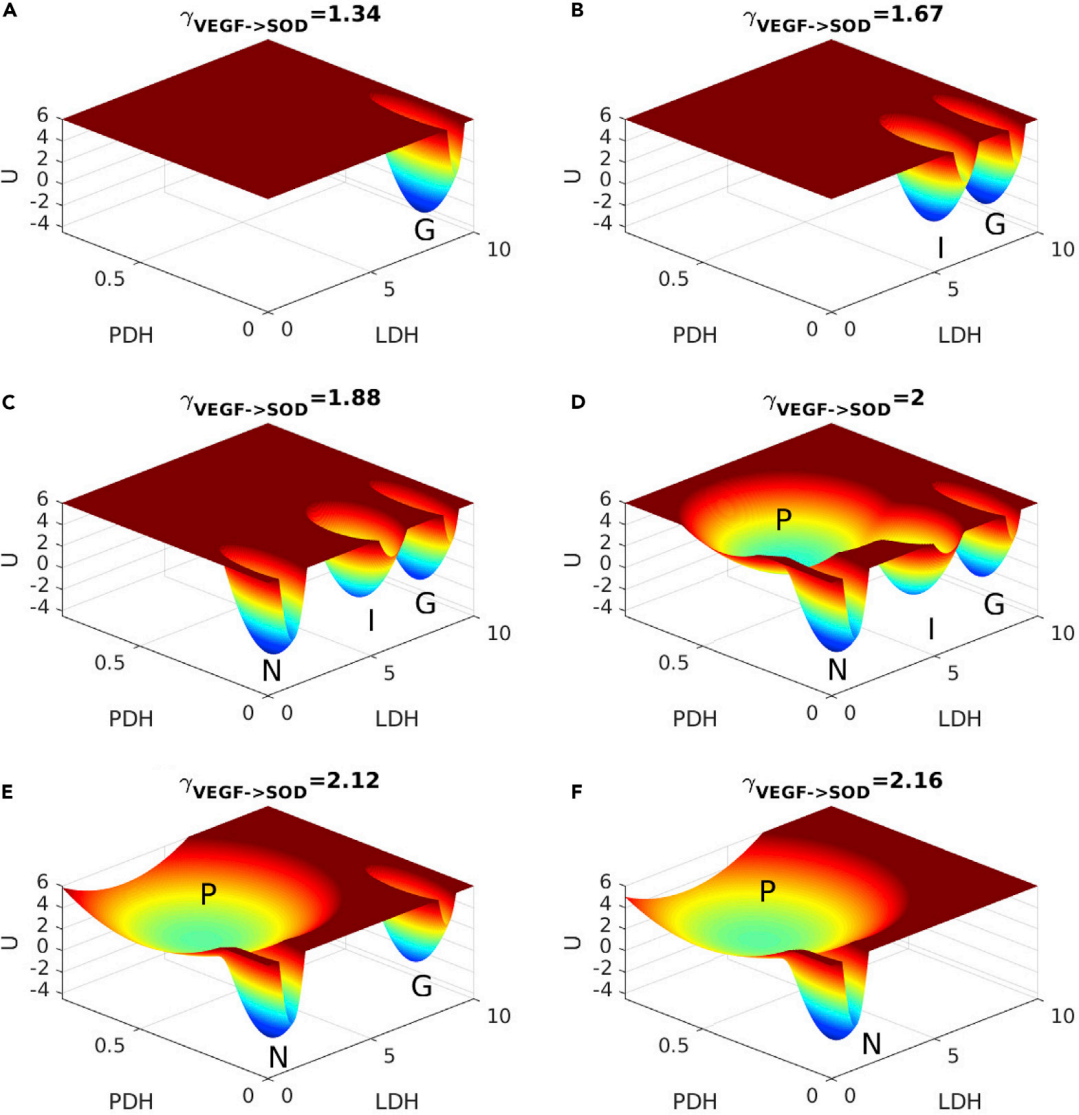
Landscape Topography Changes upon Increases in Regulation $\gamma_{VEGF->SOD}$

Different types of cancer cells are located in different organs and depend on different microenvironments. Thus, in the realistic conditions, the underlying gene and metabolic regulation strengths might be varied. To understand this further, we explored the changes of landscape topography upon the changes in important regulation strengths. Here, we change the gene-gene regulations VEGF->SOD as an example; the landscape topography changes by other important regulations are showed in Figures 4, S4, and S5. In previous experiments, the VEGF can induce SOD mRNA and the proteins in human endothelial cells (Abid et al., 2001). SOD can catalyze the dismutation of ROS into either O_2_ or H_2_O_2_. Thus, it promotes ROS clearance, when the strength of VEGF->SOD is enhanced. The landscape topography changes upon the increase of the VEGF->SOD regulation are shown in Figure 4.

At $\gamma_{VEGF->SOD}$ = 1.34, only the cancer glycolysis state emerges as shown in Figure 4A. As $\gamma_{VEGF->SOD}$ is increased to 1.67, the cancer intermediate state emerges as shown in Figure 4B. At these two regulation strengths, the system is poor for cleaning ROS. Cancer cells exhibit an increased intrinsic ROS stress, which leads to mitochondrial malfunction (Pelicano et al., 2004). Under these conditions, the system exhibits only the cancer glycolysis state or the cancer intermediate state. It is impossible to switch from the cancer state to the normal state. When $\gamma_{VEGF->SOD}$ is increased to 1.88, the ROS stress is alleviated and the normal state emerges. When the $\gamma_{VEGF->SOD}$ is increased to 2, the cancer OXPHOS state emerges. Along with the increase of $\gamma_{VEGF->SOD}$, the cancer intermediate state moves toward the cancer OXPHOS state. Then the cancer intermediate state and cancer OXPHOS state merge together. The system becomes tri-stable as shown in Figure 4E. If we reverse the process, the original cancer OXPHOS state splits into the cancer OXPHOS state and the intermediate state. This can explain why some cancer types exhibit glycolysis at the early stage and mixed cancer type at the late stage, such as prostate cancer (Costello et al., 2005). Similar landscape topography changes are shown in Figure S6, as the regulation strength of $\gamma_{HIF-1->GPI}$ is increased. When the regulation strength $\gamma_{VEGF->SOD}$ continues to increase, the cancer glycolysis state disappears as shown in Figure 4F. In the whole process, the landscape topography changes from the cancer glycolysis state to the coexistence of the normal state and cancer OXPHOS state. Although the cancer glycolysis state is destroyed by increasing $\gamma_{VEGF->SOD}$, the OXPHOS cancer state eventually emerges. This indicates the complexity of cancer metabolic mechanism and the difficulty for treating cancer.

It is worthy to mention, the glycolysis cancer state and the intermediate cancer state coexist in certain conditions as shown in Figure 4B. Regulation strengths can be perturbed by drug therapy, and this could lead to the lower expression levels of the cancer markers. However, owing to the lack of the normal state under these conditions, the cancer can come back to one of the cancer states after stopping the drugs, if the regulation strengths are not influenced significantly during the therapy.

### Landscape Changes of Normal Cell Glycolysis Switch through Decreasing O_2_ Level

**Figure 5:**
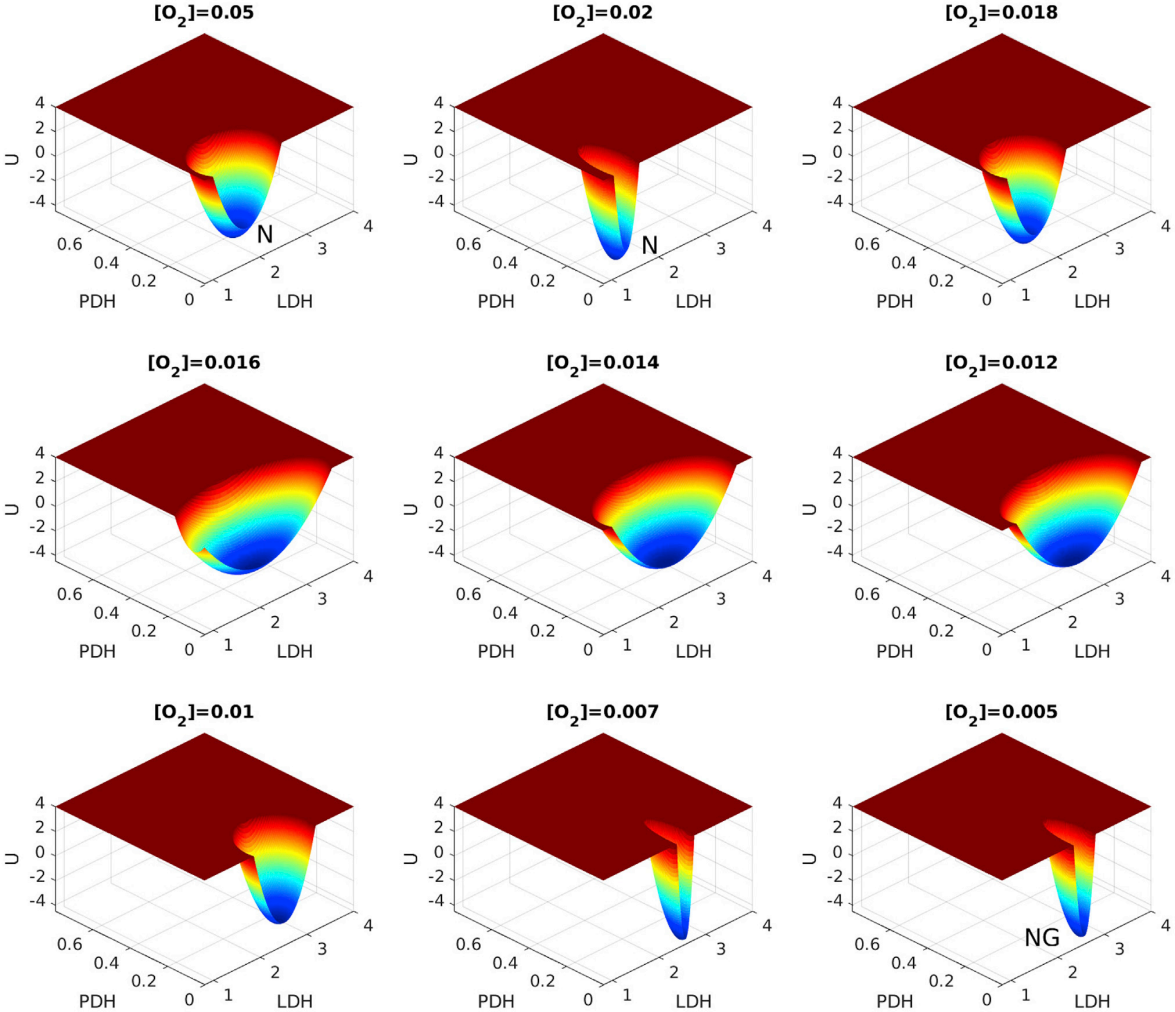
Landscape Change upon Glycolysis Switch of Normal Cells by Decreasing O_2_ Level N, normal state; NG, normal glycolysis state.

Cancer cells can generate ATP through glycolysis. However, normal cells also utilize glycolysis for generating ATP in specific conditions or developmental stages, such as hypoxia or embryogenesis. To reveal the different characteristics between cancer cells and normal cells, we depict the landscape changes of normal cells by decreasing the O_2_ level. We showed the landscape of normal cell at normal O_2_ level([O_2_] = 0.05) as shown in Figure 5. The level of LDH and PDH is low. It stays at the normal state. When the O_2_ level is decreased to 0.02, the landscape starts to become shallower and the diameter of the basin expands along the LDH axis significantly. Simultaneously the LDH level increases slowly. This means that the glycolysis of the normal cells increases and becomes less stable. When the O_2_ level is decreased to 0.01, the landscape basin becomes deeper and the diameter of the basin shrinks along the LDH axis significantly. The basin state becomes more stable. When the level of O_2_ level is decreased to 0.005, the level of LDH is near to that of the cancer glycolysis state (G). Finally, the normal glycolysis state (NG) emerges, which is distinct from the cancer glycolysis state (G).

Although the glycolysis state can be reached in cancer cells and normal cells in hypoxia, the characteristics are different. We decrease the regulation strength VEFG->SOD to view the landscape change as shown in Figure S7. When VEFG->SOD is increased to 1.5, the cancer glycolysis state emerges. For cancer cells, it has to go over the barrier to reach the cancer glycolysis state (G) and the cancer cell can have the glycolysis function. The switching to glycolysis is difficult. But once the barrier is crossed over, the switching will be fast. The reverse switching is also difficult. Conversely, for normal cells, switching from the normal state (N) to the normal glycolysis state (NG) can be realized by decreasing the O_2_ level without barrier crossing. When the O_2_ level becomes normal, the switching back to normal function spontaneously occurs.

### Probability Flux as a Dynamical Origin and Entropy Production as a Thermodynamic Origin of the Bifurcation from Normal State to Cancer State

Up to now, we discussed the landscape perspective of the cancer metabolism. Since the probability flux is also a driving force in addition to the landscape for the underlying cancer gene-metabolic network dynamics, we need to explore its role in cancer metabolism. For doing so, we calculate the mean probability flux and the associated nonequilibrium thermodynamic cost in terms of the entropy production rate (EPR) for glycolysis switching of normal cells and cancer cells, respectively.

We explore the bifurcation diagram for describing the changes of the states or phases for glycolysis switching for normal cells and cancer cells. As we can see, the switching between normal state and normal glycolysis state occurs at the O_2_ level of around 0.01, whereas the switching between the normal state and the coexistence of normal state and cancer glycolysis state occurs at the regulation strength VEFG->SOD of around 1.5 as shown in Figure 7A. Above the O_2_ level of around 0.01, the system is dominated with the normal state, whereas below the O_2_ level of around 0.01, the system is dominated with the normal glycolysis state. At the O_2_ level of around 0.01, the bifurcation or phase transition point between the normal state and the normal glycolysis state occurs. On the other hand, below the regulation strength VEFG->SOD of around 1.5, the system is dominated with the normal state, whereas above the regulation strength VEFG->SOD of around 1.5, the system is dominated with the coexistence between the normal state and the cancer glycolysis state as shown in Figures 7C and 7D. At the regulation strength VEFG->SOD of around 1.5, the bifurcation or phase transition between the normal state and coexistence of normal state and cancer glycolysis state occurs.

**Figure 6:**
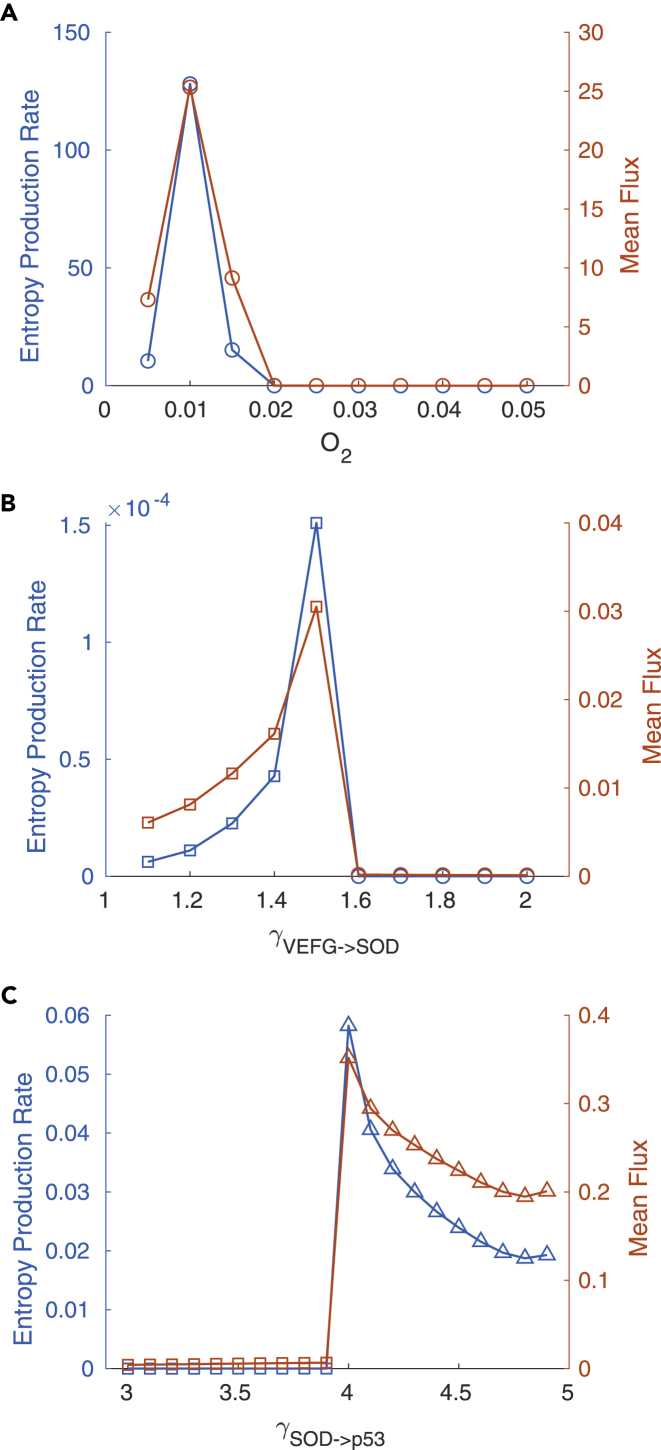
EPR and Mean Probability Flux Changes by Changing Parameters Circles: monostability (N); squares: bistability (N,G); triangles: tristability (N,G,I). (A) EPR and mean probability flux changes upon glycolysis switch of normal cells by decreasing O_2_ level. (B) EPR and mean probability flux changes upon glycolysis switch of cancer cells by regulating VEGF->SOD. (C) The EPR and probability flux change for cancer cells upon increases in regulation SOD->p53.

For normal cells, both the mean probability flux and the associate thermodynamic cost EPR are shown to be low at the ranges of O_2_ level from 0.05 to 0.02 as shown in Figure 6A. As the O_2_ level decreases further, both the mean probability flux and EPR increase sharply and then decrease sharply. Both the mean probability flux and EPR form peaks at almost the location (0.01) where the bifurcation or the phase transition appears between the normal state and the glycolysis state.

For cancer cells, both the mean probability flux and the associate thermodynamic cost EPR are low when the regulation strength VEFG->SOD is more than 1.6 as shown in Figure 6B. When the regulation strength decreases further, switching emerges from monostability to bistability, with the coexistence of the normal state and the cancer glycolysis state. The mean probability flux and EPR increase sharply and then decrease quickly with decreasing regulation strength. Both mean probability flux and EPR form peaks at almost the same location where the bifurcation or the phase transition occurs between monostability and bistability.

The landscape and probability flux have different impacts on the stability of the states. The landscape flux tends to stabilize the point attractor states due to the gradient nature of the associated force, whereas the probability flux tends to destabilize the point attractor states due to the rotational nature of the associated force. Although the different states or phases are stabilized by the landscape attractors, in order to generate new states or phases one needs to destabilize the old states and stabilize the new states. Therefore, during the switching via bifurcations or phase transitions, the probability flux becomes crucial for the changes in stabilities. In fact, the probability flux provides a dynamical origin of the bifurcation with the stability changes and state switching. The thermodynamic cost in terms of the entropy production rate is closely related to the probability flux, as illustrated in Figure 6. In steady state, the EPR is equal to the heat dissipation rate. An increase of EPR means more thermodynamic dissipation for maintaining the state. A peaked EPR suggests that the glycolysis switching process of normal cells and cancer cells require significantly more thermodynamic dissipation. In other words, to have the glycolysis switching of normal cells and cancer cells, higher mean probability flux and EPR are necessary.

**Figure 7:**
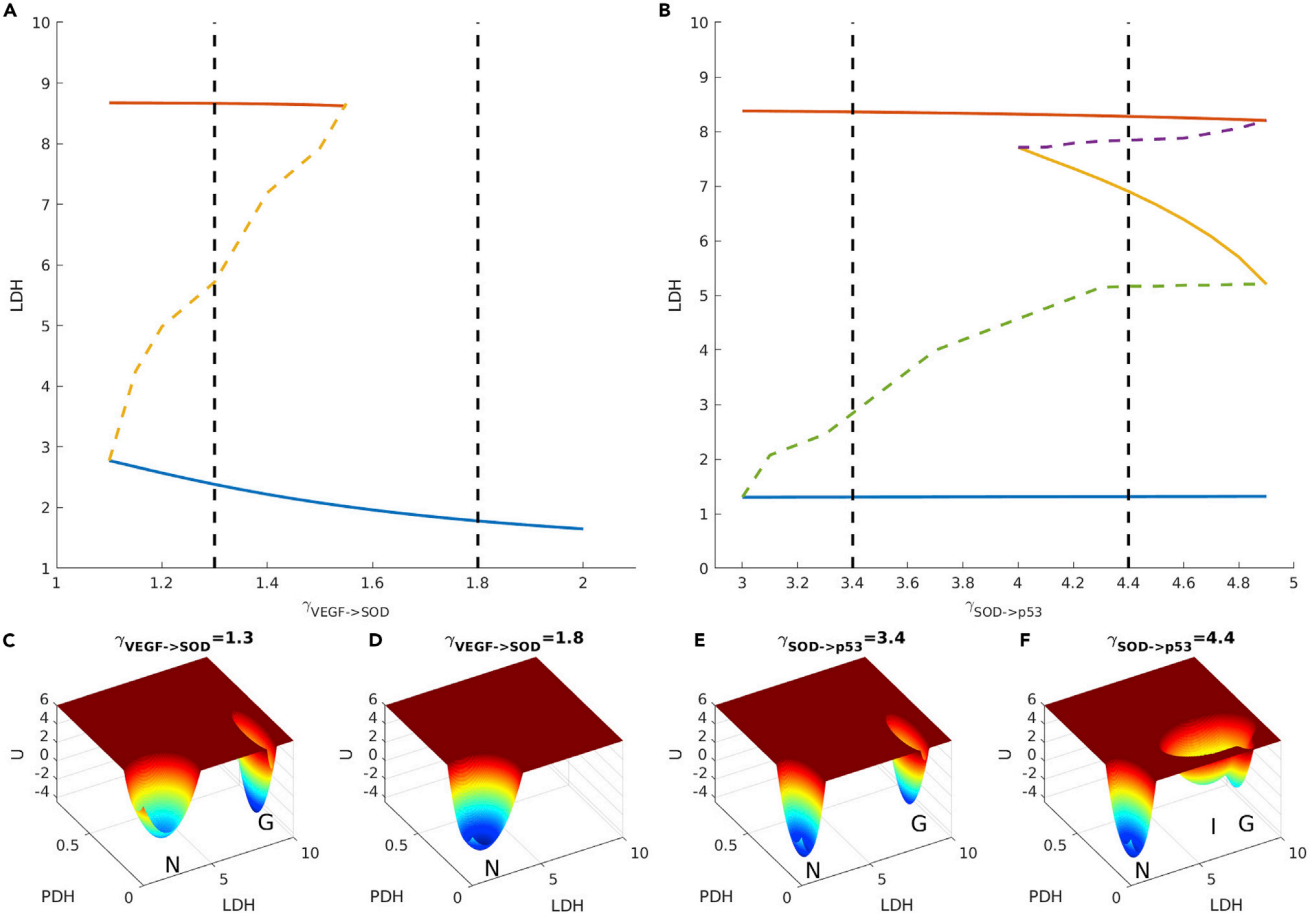
Bifurcation Diagrams and Landscape Change upon Regulating VEGF->SOD and SOD->p53 Strength (A) Bifurcation diagrams with regulation of VEGF->SOD. (B) Bifurcation diagrams with regulation of SOD->p53. (C) Landscape at $\gamma_{VEGF->SOD}$ = 1.3. (D) Landscape at $\gamma_{VEGF->SOD}$ = 1.8. (E) Landscape at $\gamma_{SOD->p53}$ = 3.4. (F) Landscape at $\gamma_{SOD->p53}$ = 4.4. Solid line: In (A) and (B), blue, red, and yellow solid lines represent normal, cancer glycolysis, and cancer intermediate steady state, respectively. Colored dashed lines represent unstable steady states.

As we can see, both the glycolysis switching of normal and cancer states have clear quantitative signatures of peaked probability flux and entropy production rate. The probability flux provides a dynamical origin of the bifurcation or phase transitions for glycolysis switching in normal and cancer cells. The entropy production rate provides the thermodynamic origin of the bifurcations or phase transitions for glycolysis switching in normal and cancer cells. Another example shows the peaked mean probability flux and EPR at the bifurcation from the bistability of normal state and cancer glycolysis state to tristability of normal state, cancer glycolysis state, and intermediate state as shown in Figures 6C, 7B, 7E, and 7F. We expect that bifurcations or phase transitions of all the processes involving cancer formation and recovery are driven by the probability flux and the associated entropy production rate. The probability flux and EPR can be quantified. They may provide an indicator or marker for cancer formation useful for early diagnosis and prevention.

### Cancer Metabolism Oscillation Landscape

**Figure 8:**
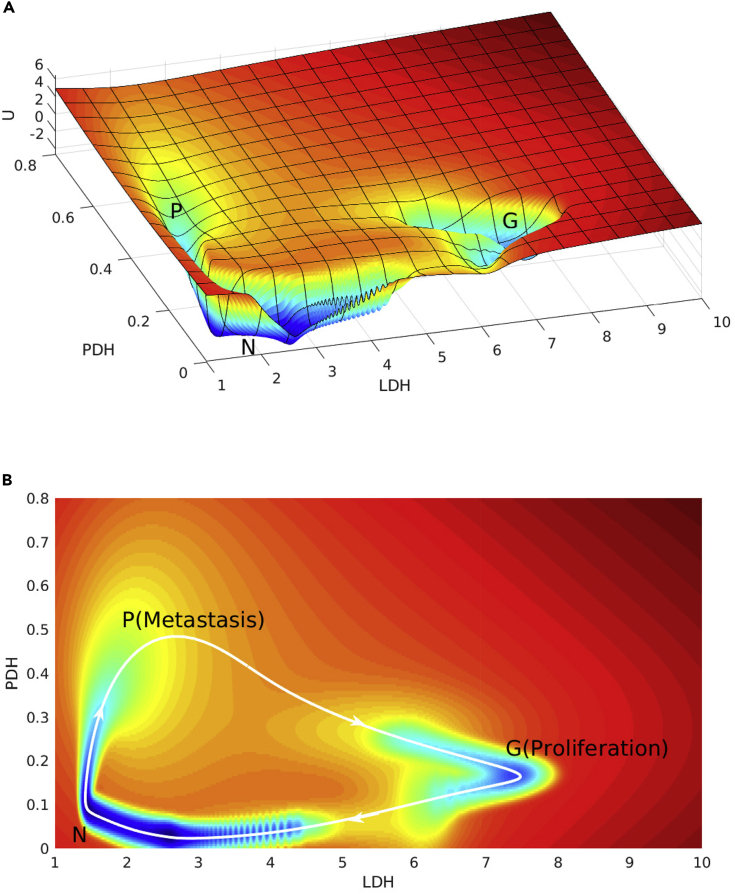
The Landscape of Cancer Metabolism Oscillation The white arrows represent the directions of cancer metabolism oscillation. (A) The landscape of cancer metabolism oscillation in 3D. (B) The landscape of cancer metabolism oscillation in 2D.

It is interesting to see that oscillation emerges under certain regulatory interactions. The glycolytic oscillations have been observed in individual HeLa cervical cancer cells (Amemiya et al., 2017). A model of HeLa cancer cells growing under hypoxic conditions shows that the oscillations persist in a wide range of parameters (Martin et al., 2017). In our study, the oscillations have been found and the corresponding landscape has a shape of inhomogeneous Mexican hat displayed in Figure 8A. Although landscape attracts the system down to the oscillation ring valley, it is the probability flux that drives the stable oscillation flow. Interestingly, the normal state, the cancer OXPHOS state, and the cancer glycolysis state attractors are deeper than the other places on the limit cycle oscillation ring. This infers the longer residence time of these states along the oscillation paths. As shown in Figure 8B, the limit cycle oscillates from the normal state to the cancer OXPHOS state and then to the cancer glycolysis state forming a clockwise cycle. Glycolysis has the advantage of producing ATP at a high rate and generating intermediate metabolites required for rapid cell proliferation (Heiden et al., 2009), whereas OXPHOS contributes significantly to the cancer metastasis (Viale et al., 2015, Roesch et al., 2013, Tan et al., 2015, Porporato et al., 2014). The limit cycle oscillation between the proliferation and metastasis illustrates serious malignancy of cancer in clinics such as systemic metastasis (Burton et al., 2008). As shown in Figure 8B, the LDH expression level decreases from the cancer glycolysis state to the normal state. The ability of proliferation descends. At this state, the cancer cells behave like normal cells. Previous studies suggest that the balance between dormancy and death of cancer cells may be mediated by the precise levels of proliferation and survival signals (Yeh and Ramaswamy, 2015). Then, the PDH expression level increases from the normal state to the cancer OXPHOS state. The ability of metastasis ascends. The LDH expression level increases and PDH expression level decreases from the cancer OXPHOS state to the cancer glycolysis state. Metabolic oscillation of cancer cell can lead to the switching of proliferation and metastasis of cancer cell physiologically. Once cancer cells emerge and reach a certain amount, the nutrition in the microenvironment is limited to support cancer cells. For survival, cancer cells with metabolic oscillation promote metastasis, when it stays at the OXPHOS state in the limit cycle. However, to acquire the ability for metastasis, cancer cells need required mutations, which may happen at the glycolysis state in the limit cycle. Once the required conditions mature and cancer cells stay at the OXPHOS state in the limit cycle, they may start to metastasize. Cancer cells can colonize at any place good enough for cancer cells to survive for a long time in the oscillation. The switching between proliferation and metastasis demonstrates the great malignancy of cancer. It has been reported that certain lung cancers metastasize quickly to multiple sites (Chaffer and Weinberg, 2011).

**Figure 9:**
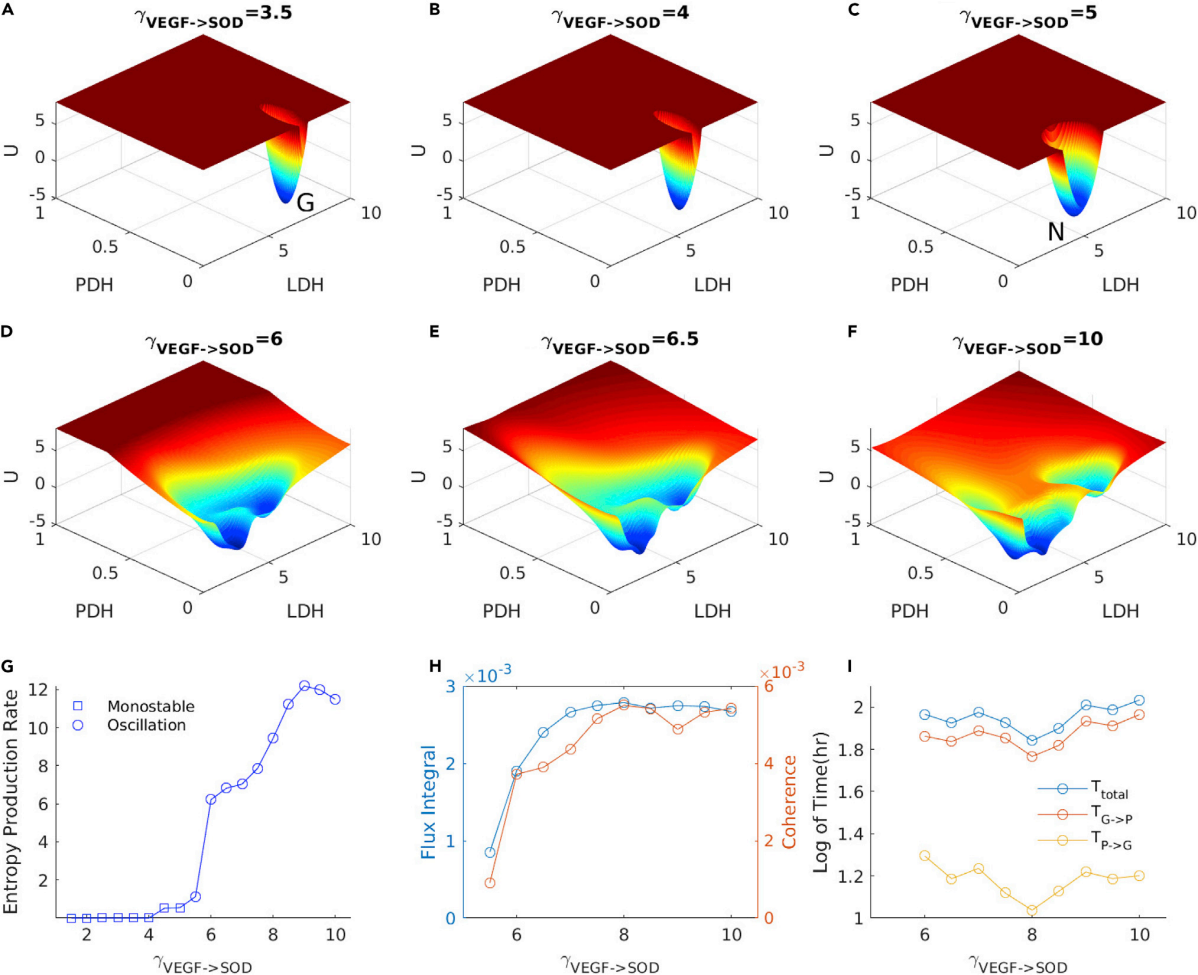
The Landscape Topography of Oscillation Changes upon the Increase of Regulation of $\gamma_{VEGF->SOD}$ (A and B) Emergence of cancer glycolysis state with $\gamma_{VEGF->SOD}$ from 3.5 to 4. $\gamma_{VEGF->SOD}$ = 3.5(A). $\gamma_{VEGF->SOD}$ = 4(B). (C) Emergence of normal state with $\gamma_{VEGF->SOD}$ = 5. (D–F) Emergence of cancer metabolism oscillation with $\gamma_{VEGF->SOD}$ from 6 to 10. $\gamma_{VEGF->SOD}$ = 6(D). $\gamma_{VEGF->SOD}$ = 6.5(E). $\gamma_{VEGF->SOD}$ = 10(F). (G) Entropy production rate of monostability and oscillation. (H) Probability flux integral and coherence of oscillation. (I) Switching time between glycolysis and OXPHOS. *T*_*total*_: oscillation time; $T_{G->P}$: switching time from G to P; $T_{P->G}$: switching time from P to G.

We also quantified the changes of the oscillation landscape topography upon the changes of the regulation strength of VEGF−>SOD. As shown in Figure 9, we increase the regulations of VEGF−>SOD starting from the monostable state, cancer glycolysis state. As the VEGF−>SOD regulation strength increases, the expression level of LDH decreases. When $\gamma_{VEGF->SOD}$ is increased to 5, the system changes from the original glycolysis cancer state to the normal state. The limit cycle emerges when $\gamma_{VEGF->SOD}$ is increased to 6. As the regulation strength $\gamma_{VEGF->SOD}$ increases further, the limit cycle expands. The maximum expression levels of LDH and PDH are increased. When $\gamma_{VEGF->SOD}$ is increased to 10, the maximum expression levels of LDH and PDH reach to the expression levels of the cancer glycolysis state and the cancer OXPHOS state, respectively. The limit cycle oscillates coherently among the normal state, the cancer OXPHOS state, and the cancer glycolysis state.

We also calculate the probability flux integral as a measure of the magnitude of the probability flux and the coherence of the oscillation when the oscillation emerges as shown in Figure 9H. The probability flux integral correlates with the coherence. This indicates that the higher probability flux leads to higher oscillation coherence or the stability of the oscillation flow. The probability flux integral and coherence are increased sharply when the system switches from the original normal state phase to the oscillation phase. This indicates that a larger probability flux leads to a stronger driving force for the tendency of the oscillation. In addition, we calculate the entropy production rate (EPR) for the phase transition from monostability to oscillation by increasing the regulation strength of VEGF−>SOD as shown in Figure 9G. The EPR represents the total entropy production rate. In steady state, the EPR is equal to the heat dissipation rate. Therefore, increasing of EPR means more dissipation for maintaining the state. When the system switches to the oscillation, the EPR is sharply increased. This implies that the oscillation phase requires much more energy cost to maintain. Therefore, more energy has to be pumped into the system for switching to the oscillation. This also implies that cancer metabolism oscillation consumes more energy in order to support the malignancy of cancer. We notice that both probability flux integral and EPR have a sharp increase at the regulation strength of VEGF−>SOD around 6. This is also near the bifurcation point from the monostable normal state to the oscillation as seen in the bifurcation diagram. This again illustrates that the probability flux provides a dynamical origin and EPR provides a thermodynamic origin for the emergence of the bifurcation or phase transition to the oscillation. Besides, we also calculate the oscillation and switching times between glycolysis and OXPHOS as shown in Figure 9I. The switching time from glycolysis to OXPHOS is much greater than from OXPHOS to glycolysis. The oscillation time is determined by both the flux and the circumference in the limit cycle. The timescale of the oscillation is much greater than the oscillation timescale in the previous experimental studies (Amemiya et al., 2017). The oscillation of glycolysis in the previous experimental studies is damped and caused by the sudden change of microenvironment such as the starvation of cells. It is based on the short time period regulations between genes and metabolites. The oscillation in our model is stable, and the model is focused on the studies of steady characteristics of cancer cells. It is based on the long time period regulations between genes and metabolites.

### Metabolic Therapeutic Target Prediction

The metabolic characteristics of cancer cells are different from those of the normal cells. Targeting on cellular metabolism is a promising strategy for cancer therapy (Zhao et al., 2013). Here, we predict the metabolic therapeutic targets based on the landscape analysis. For each gene or enzyme $x_{i}$, $F\left(x_{i}\right)$ is changed to $F\text{'}\left(x_{i}\right)=F\left(x_{i}\right)+c_{i}$. The term $c_{i}$ represents the corresponding changes in activation or inhibition regulations due to the perturbations on the variable. The potential landscape of the four steady-state attractors are quantified for the corresponding $c_{i}$, respectively. If $c_{i}>0$, it represents the activation of the gene or the enzyme. If $c_{i}<0$, it represents the inhibition of the gene or the enzyme. We define the changes of the cancer OXPHOS state as the degree of therapeutic effect on the OXPHOS cancer type and the changes of the cancer glycolysis state as the degree of therapeutic effect on the glycolysis cancer type. If the changes of the barrier height are negative, this leads to the instability of certain cancer steady state. This represents the positive effect on the therapeutic target.

**Figure 10:**
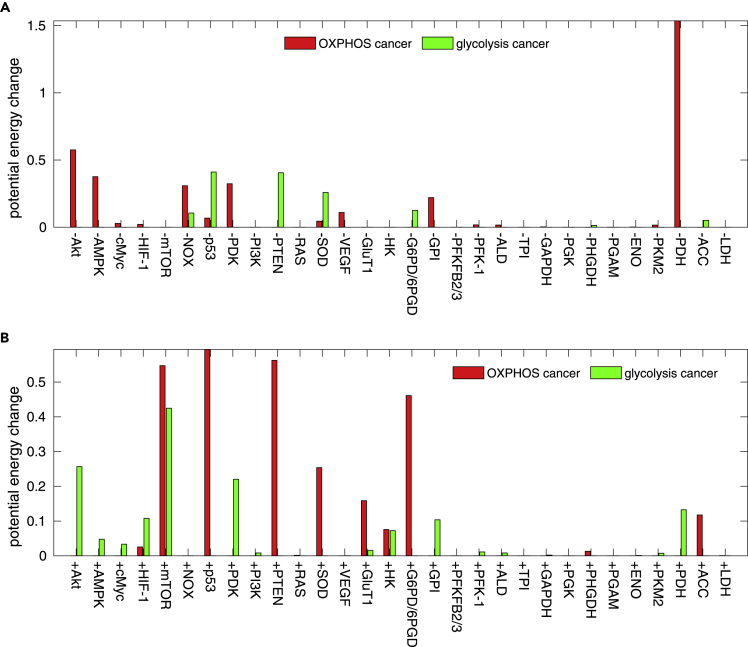
Predictions of Metabolic Therapeutic Targets for OXPHOS Cancer and Glycolysis Cancer The parameter c_*i*_ = 2 × 10^−4^. (A) Therapeutic effect for inhibiting the expressions of the genes and the enzymes. (B) Therapeutic effect for promoting the expressions of the genes and the enzymes.

The effects of metabolic therapeutic target are shown in Figure 10. The gene or the enzyme name starting with ‘-’ means the inhibition in the expressions of the therapeutic target, and the one starting with ‘+’ means the activation in the expressions of the therapeutic target. We predict five important OXPHOS cancer therapeutic targets, -PDH, +p53, +mTOR, +PTEN, and -Akt, and three important glycolysis cancer therapeutic targets, -PTEN, -p53, and +mTOR. Melanoma belongs to the OXPHOS cancer type. It is reported that the suppression of PDH phosphorylation leads the melanoma cells to death *in vitro* (Kaplon et al., 2013). Inhibition of Akt expression converts the melanoma cells to be less invasive (Govindarajan et al., 2007). What is interesting is that +mTOR is both an OXPHOS cancer therapeutic target and a glycolysis cancer therapeutic target.

It has been reported that combined therapies give more effectiveness on cancer metabolism than the individual therapy (Sahra et al., 2010, Cheong et al., 2011). Thus, we predicted the effects of combination therapy. This is according to the landscape topography changes in terms of the barrier heights, which lead to the higher stability/lower stability for the cancer basins of attraction. The combination therapy for OXPHOS and glycolysis cancer is predicted in Figures S8 and S9. The values in the color matrix represent the degrees of therapeutic effect. The red color represents the positive therapeutic effect, whereas the blue color represents the negative therapeutic effect. The most effective combinations of therapy for the OXPHOS cancer type are +GluT1 and -PKM2. The most effective combinations of therapy for glycolysis cancer type are +mTOR and +NOX.

For the malignancy of cancer metabolism oscillation, we aim to weaken the oscillation capability of the limit cycle and drive the system to become monostable at the normal state by promoting or inhibiting certain genes or enzymes. The oscillation capability can be estimated by the barrier height from the highest point at the center island to the lowest point on the limit cycle. We predict the effect of the therapeutic target for cancer oscillations as shown in Figure S10. The most effective therapeutic targets are +mTOR, +PTEN, and +PDH.

## Discussion

Reprogramming of cellular metabolism for cancer cell is complex. It is still challenging to reveal the underlying mechanism for influencing the biological function of the cancer cells by metabolism variation. In this study, the cancer gene-metabolism integrative network model was built including 13 genes, 17 enzymes, and 23 metabolites. The network includes metabolic reactions of the glycolysis pathway and TCA cycle. In order to reflect the realistic metabolic pathway, we include the whole network on cancer metabolism for modeling. Four steady-state basins of attractors emerge, including the normal state (N), the cancer OXHPOS state (P), the cancer glycolysis state (G), and the cancer intermediate state (I). It is interesting that the three cancer states correspond to the oxidative-phosphorylation-dependent cancer cell type, the glycolysis-dependent cancer cell type, and the mixed cancer cell type. These cancer types have been observed in the experiments, respectively (Amann and Hellerbrand, 2009, Calvisi et al., 2011, Elia et al., 2015, Graziano et al., 2016, Haber et al., 1998, Ma et al., 2013, Obre and Rossignol, 2015).

In the realistic biological environment, the regulation/interaction strengths might not be fixed. We explore the changes of landscape topography upon changes of important regulation strengths. The cancer cells in different organs or microenvironments exhibit different characteristics. For example, the oxidative-phosphorylation-dependent cancer type such as melanomas and glioblastomas resides at certain location on the landscape shown in Figure 4B, owing to its microenvironment. In a similar way, the glycolysis-dependent cancer type resides at another location on the landscape shown in Figure 4F. Besides, cancer cells can switch metabolism for ATP generation such as prostate cancer at different stages (Costello et al., 2005). This can be explained by the changes of the landscape topography in Figures 4D and 4E. Regulation/interaction strengths of each patient cancer cells are different owing to individual conditions. This implies that a patient can relapse if the regulation strengths are not influenced significantly during the therapy as shown clearly on the landscape. However, different patients with different regulation strengths can have different therapeutic results shown on the landscape topography in Figure 4F. Thus, the landscape topography provides a framework to study specific cancer types, specific cancer stages, and specific individual patients.

Both cancer cells and normal cells can depend on glycolysis for generating ATP at specific conditions. Some types of cancer cells can generate energy through glycolysis, even at the microenvironment containing enough O_2_. Normal cells have to switch to glycolysis owing to hypoxia. The glycolysis switching for normal and cancer cells can be described by the bifurcation. The glycolysis switching process or bifurcation shows the peaks in both the mean probability flux and entropy production rate. We also calculate the mean probability flux and EPR for another bifurcation and obtain similar results as shown in Figures 7B and 6C. It shows that the peak of mean probability flux and EPR appears near the bifurcation point. The bifurcation leads to the emergence of the cancer states (G,I,P) and gives rise to the possibility of appearance of cancer cells. The mean probability flux provides a dynamical origin of the bifurcation, whereas EPR provides a thermodynamic origin of the bifurcation. The cell metabolic and cancer states can be measured through Seahorse analyzer Fluorescence-lifetime imaging microscopy (Jia et al., 2020, Lukina et al., 2019). The heat change of cells can be measured through isothermal titration calorimetry (Rodenfels et al., 2020). However, these may not be quantitatively accurate enough to clearly distinguish different states, although these methods can be used to measure the metabolic change qualitatively or semi-quantitatively. Thus, there seems no obvious quantitative boundary between the cell metabolic states and cancer states. In other words, these measurements usually give continuous values and it is not clear if there is a clear separation between the cell metabolic states and cancer states. There seems no other clear indicators from the perspective of biology for seeing the emergence of the cancer and cell metabolic states. In contrast, from the physics perspective, we found that there is an obvious peak of the nonequilibrium driving force for dynamics in terms of the mean probability flux and the nonequilibrium thermodynamic cost in terms of the entropy production rate EPR to clearly indicate the emergence of different states, the switching process between these states, and the corresponding bifurcations/phase transitions based on the landscape and flux theory. Therefore, the physical measures in terms of nonequilibrium dynamics and thermodynamics can provide quantitative predictors and indicators for clearly seeing the emergence of the cell states such as cancer and the cell metabolic states as well as the corresponding switching in terms of bifurcations/phase transitions between them. The mean probability flux and the EPR can provide as a quantitative indicator or marker for the emergence of cell metabolic states and cancer states useful to the early diagnosis and prevention.

Cancer metabolism oscillations emerge upon certain cell-cell regulations/interactions. The glycolytic oscillations have been observed in individual HeLa cervical cancer cells (Amemiya et al., 2017). In our study, we found that the cell can oscillate between the cancer glycolysis state, normal state, and cancer OXPHOS state clockwise. Along with the oscillation, the cell can switch between proliferation and metastasis. This can lead to serious malignancy of cancer in clinics (Burton et al., 2008). Compared with other cases, cancer metabolism oscillation is more dangerous for patients. Through the analysis of the probability flux integral and coherence as well as entropy production rate, it is suggested that more energy is dissipated to support the malignancy of cancer.

We predict five most effective metabolic therapeutic targets for the OXPHOS cancer type and three most effective metabolic therapeutic targets for the glycolysis cancer type. We suggest that +mTOR is effective for both the OXPHOS cancer type and the glycolysis cancer type. Combinations of therapy have been suggested to be more effective (Sahra et al., 2010, Cheong et al., 2011). We predict the most effective combination for OXPHOS cancer type as +GluT1 and -PKM2 and the one for glycolysis cancer type as +mTOR and +NOX, respectively. We also predict three most effective metabolic therapeutic targets for cancer oscillation as +mTOR, +PTEN, and +PDH.

In summary, our model provides insights into the metabolism of different cancer types. The landscape and probability flux approach provides a framework for studying the underlying mechanism of the specific cancer type, specific cancer stage, and specific individual. The probability flux and associated entropy production rate provide, respectively, a dynamic and thermodynamic origin as well as a quantitative indicator for the bifurcations or phase transitions of cell switching and cancer formation. The cancer metabolism oscillation uncovered in our model brings a perspective on how malignant cancer cells switch between proliferation and metastasis. The predicted metabolic therapeutic targets maybe useful in developing anti-cancer strategies.

### Limitations of the Study

The metabolism of cancer cell is complex. Cancer cell can utilize multiple ways to acquiring nutrient. Besides uptake of glucose, cancer cells can swallow proteins, living cells, and apoptotic bodies, which are resolved into small molecules as nutrient for metabolism (Pavlova and Thompson, 2016). The model in our study is not able to include these kinds of complex function of cancer metabolism. Besides the hallmark of metabolism reprogramming, cancer involves other complex hallmarks (Hanahan and Weinberg, 2011). Although the model in our study contains many important genes, it is difficult to include all the genes for all the hallmarks of cancer, owing to the intrinsic complexity. A comprehensive network including all cancer hallmarks is still very challenging. We provide the metabolic therapeutic targets for cancer based on our current model and the landscape-flux theory. The differences in cancer cell microenvironments between tissues can give rise to the differences in the metabolism and gene regulation strengths of cancer cells. Owing to the lack of the precise measurements of the model parameters for different tissues, the effect of cancer metabolic therapeutic target predicted may vary in different tissues. The predicted cancer metabolic therapeutic targets can still be challenging for precise treatment.

## Methods

All methods can be found in the accompanying Transparent Methods supplemental file.

## Data and Code Availability

All code are given in GitHub https://github.com/lwb422/cancer_metabolism.

## Supplemental Information

Document S1. Transparent Methods, Figures S1–S10, and Tables S1–S6
