# Supplementary material for: Uncovering the Underlying Mechanisms of Cancer Metabolism through the Landscapes and Probability Flux Quantifications
Source: iScience. 2020 Mar 25;23(4):101002. doi: 10.1016/j.isci.2020.101002 (PMC7150521; doi:10.1016/j.isci.2020.101002)
Supplement: Document S1. Transparent Methods, Figures S1–S10, and Tables S1–S6 [file mmc1.pdf]

**iScience, Volume *23***

## Supplemental Information

## Uncovering the Underlying Mechanisms of Cancer Metabolism through the Landscapes and Probability Flux Quantifications

**Wenbo Li and Jin Wang**

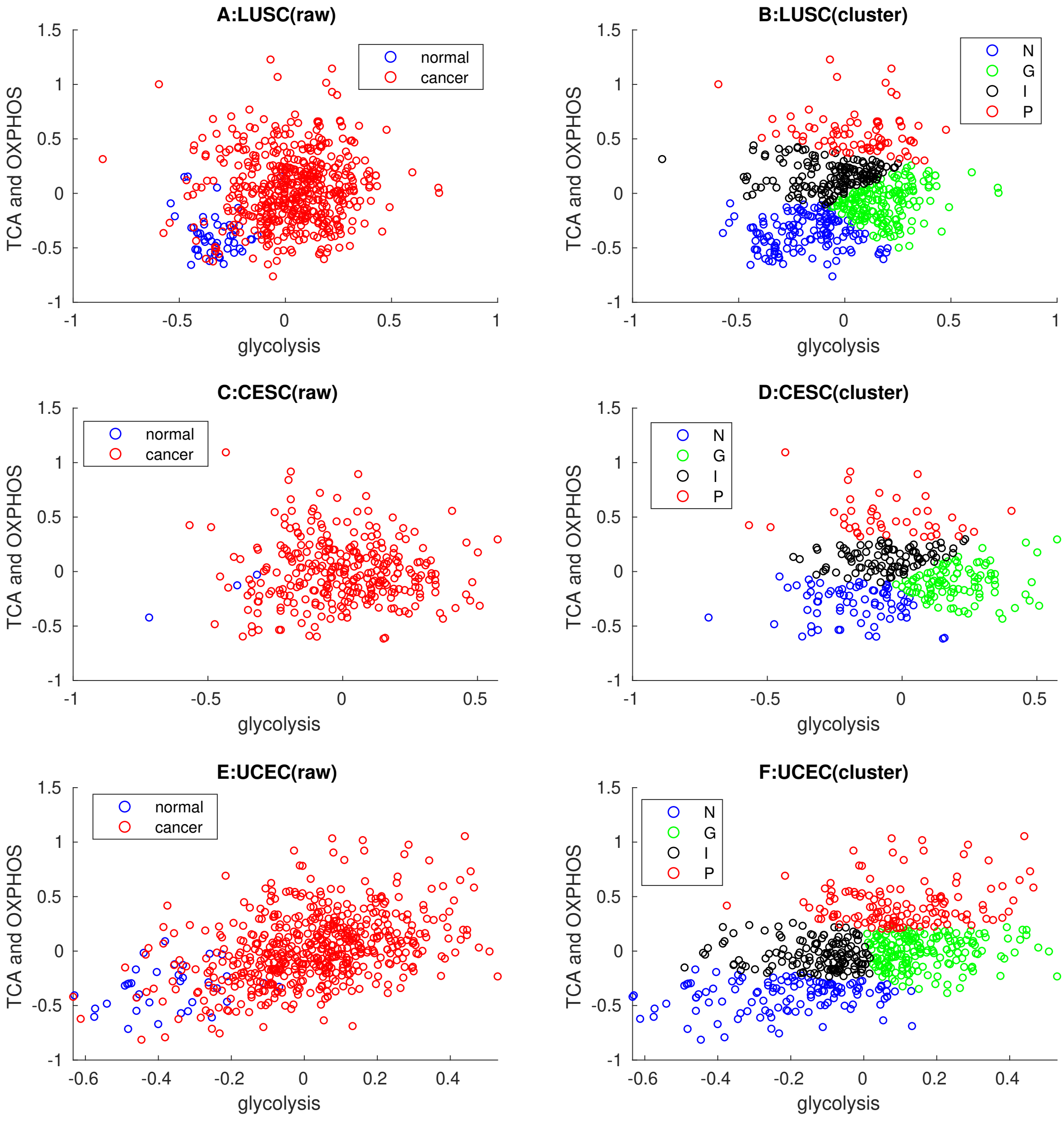


Figure S1: Glycolysis, TCA cycle and OXPHOS related gene expressions from GDC (related to Figure 2). (A,C,E)Gene expression data with normal and cancer samples. (B,D,F)Gene expression data clustered by K-means. LUSC: lung squamous cell carcinoma; LUAD: lung adenocarcinoma; UCEC: uterine corpus endometrial carcinoma. N: normal state; P: cancer OXPHOS state; G: cancer glycolysis state; I: cancer intermediate state.

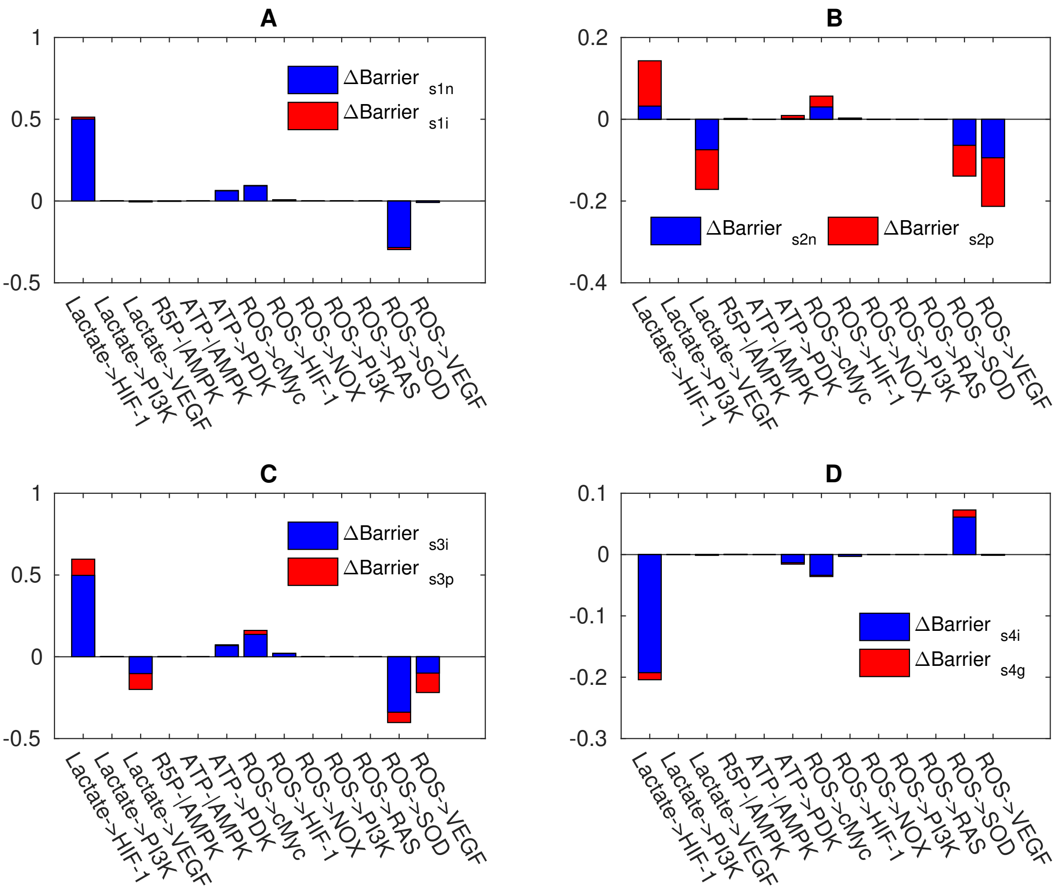


Figure S2: Global sensitivity analysis for the 13 metabolite-gene regulations (related to Figure 3). X-axis represents the 13 metabolite-gene regulations. Y-axis represents the barrier changes. Each parameter is increased by 1% individually. $\Delta\text{Barrier}_{s1n}$: the change of the barrier from s1 to normal steady state. $\Delta\text{Barrier}_{s1i}$: the change of the barrier from s1 to cancer intermediate state. $\Delta\text{Barrier}_{s2n}$: the change of the barrier from s2 to normal state. $\Delta\text{Barrier}_{s2p}$: the change of the barrier from s2 to cancer OXPHOS state. $\Delta\text{Barrier}_{s3n}$: the change of the barrier from s3 to cancer normal state. $\Delta\text{Barrier}_{s3i}$: the change of the barrier from s3 to cancer intermediate state. $\Delta\text{Barrier}_{s4i}$: the change of the barrier from s4 to cancer intermediate state. $\Delta\text{Barrier}_{s4g}$: the change of the barrier from s4 to cancer glycolysis state.

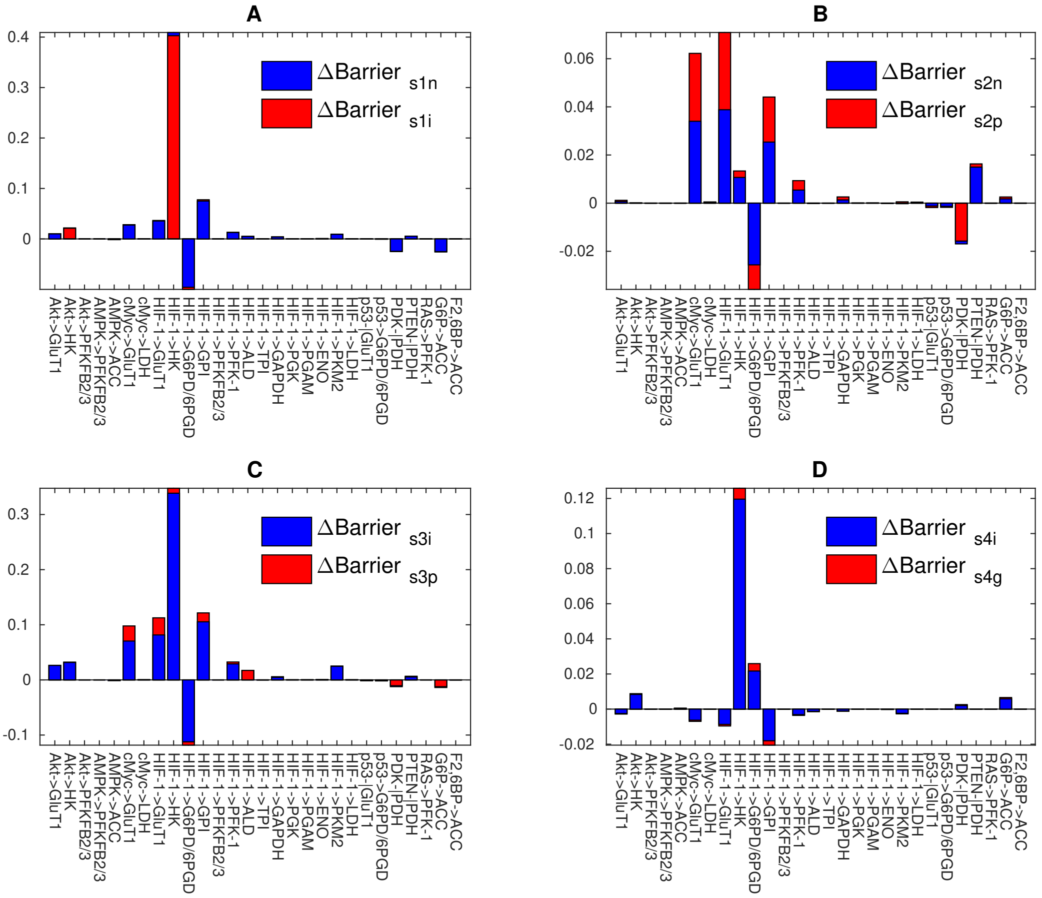


Figure S3: Global sensitivity analysis for the 28 gene-enzyme regulations (related to Figure 3). X-axis represents the 28 gene-enzyme regulations. Y-axis represents the barrier changes. Each parameter is increased by 1% individually. $\Delta\text{Barrier}_{s1n}$: the change of the barrier from s1 to normal steady state. $\Delta\text{Barrier}_{s1i}$: the change of the barrier from s1 to cancer intermediate state. $\Delta\text{Barrier}_{s2n}$: the change of the barrier from s2 to normal state. $\Delta\text{Barrier}_{s2p}$: the change of the barrier from s2 to cancer OXPHOS state. $\Delta\text{Barrier}_{s3n}$: the change of the barrier from s3 to cancer normal state. $\Delta\text{Barrier}_{s3i}$: the change of the barrier from s3 to cancer intermediate state. $\Delta\text{Barrier}_{s4i}$: the change of the barrier from s4 to cancer intermediate state. $\Delta\text{Barrier}_{s4g}$: the change of the barrier from s4 to cancer glycolysis state.

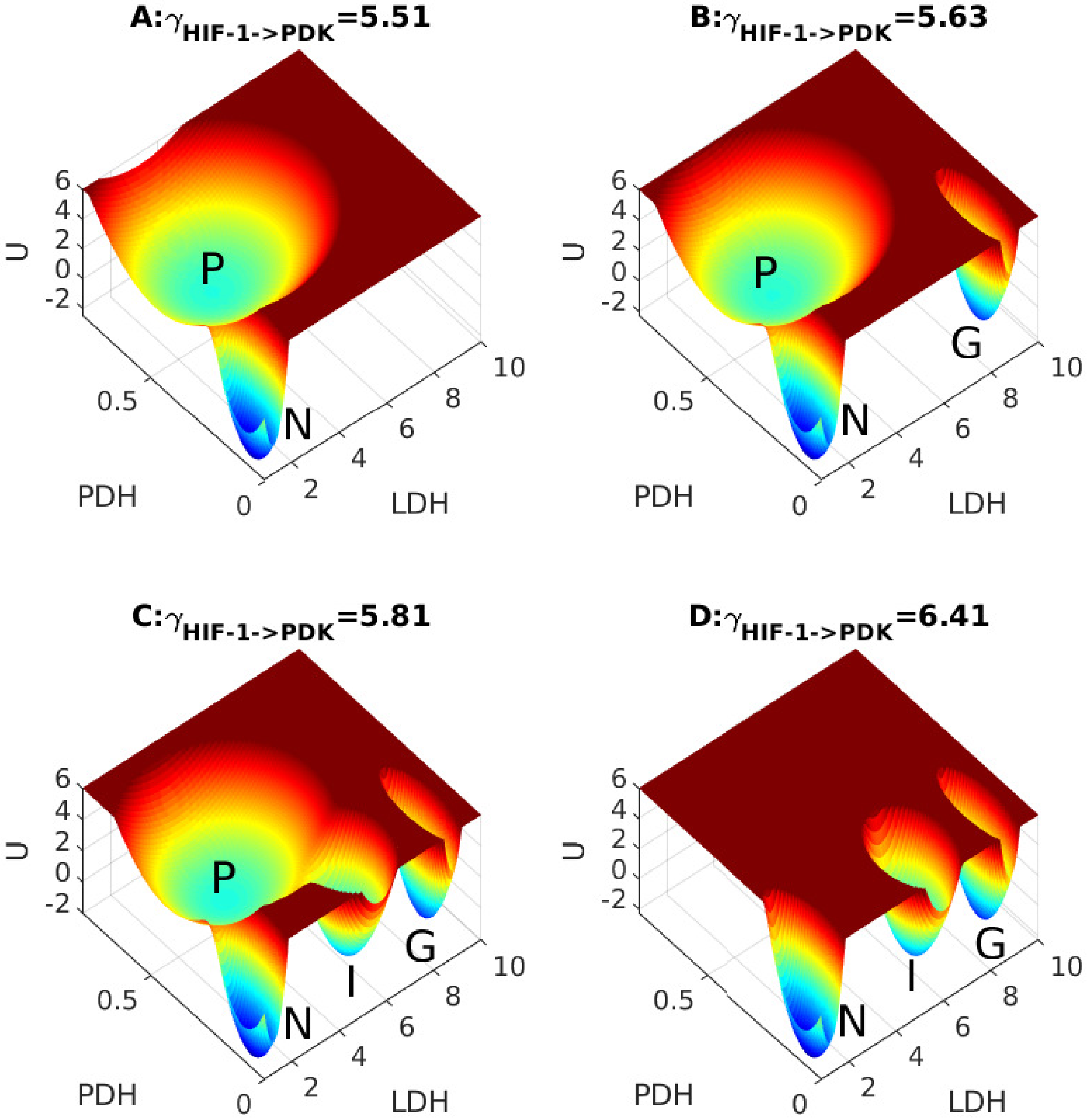


Figure S4: Landscape topography changes upon increases in regulation $\gamma_{HIF-1->PDK}$ (related to Figure 4).

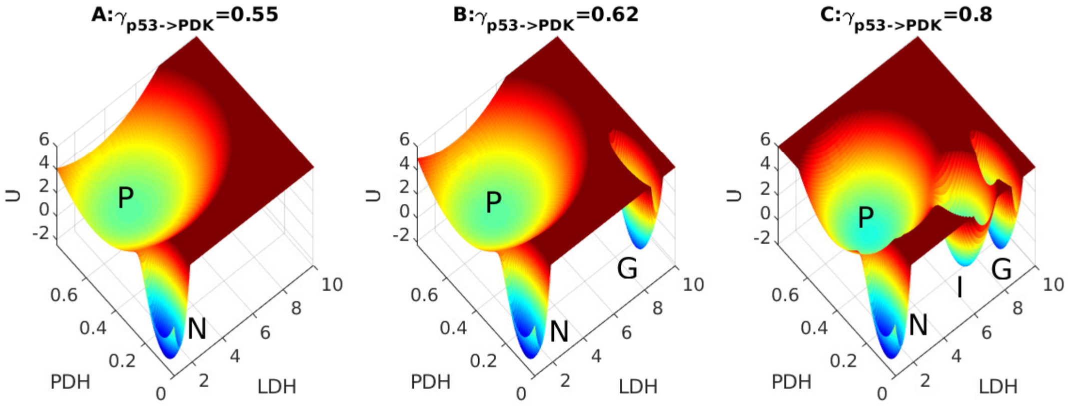


Figure S5: Landscape topography changes upon increases in regulation $\gamma_{P53->PDK}$ (related to Figure 4).

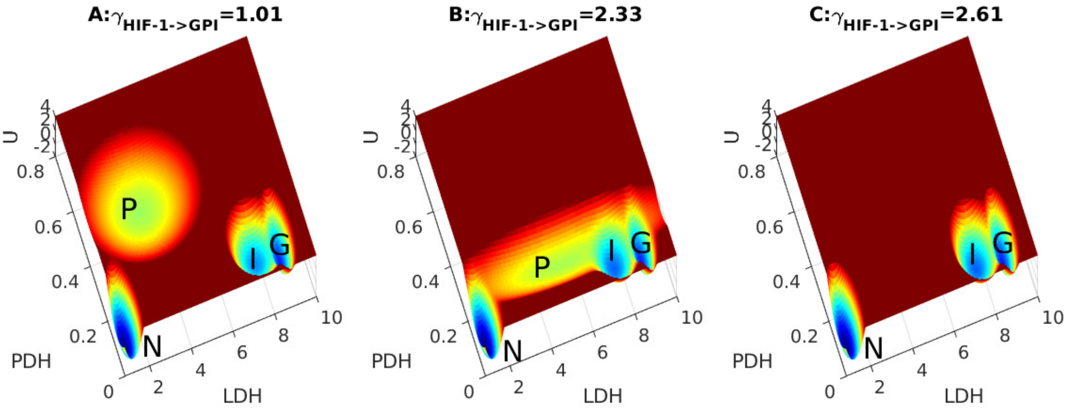


Figure S6: Landscape topography changes upon increases in regulation $\gamma_{HIF-1->GPI}$ (related to Figure 4).

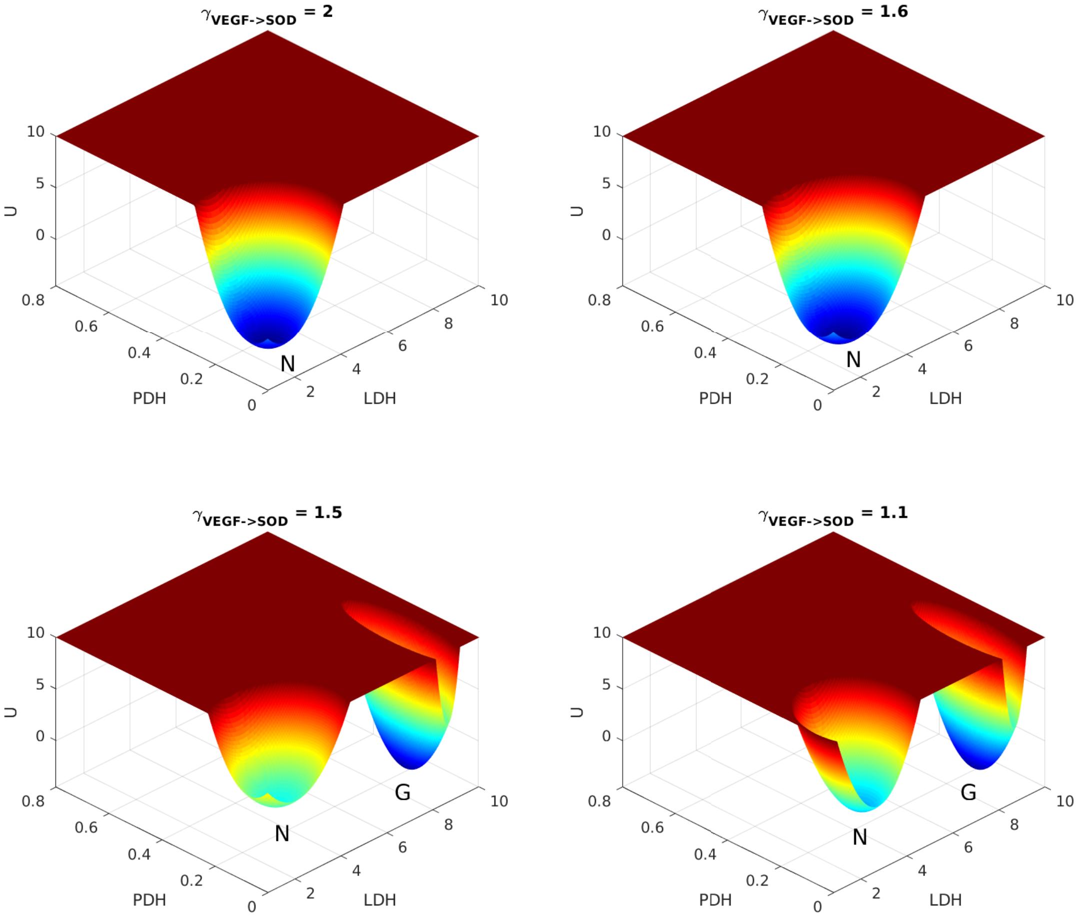


Figure S7: Landscape topography changes upon increases in regulation $\gamma_{VEGF->SOD}$ (related to Figure 6).

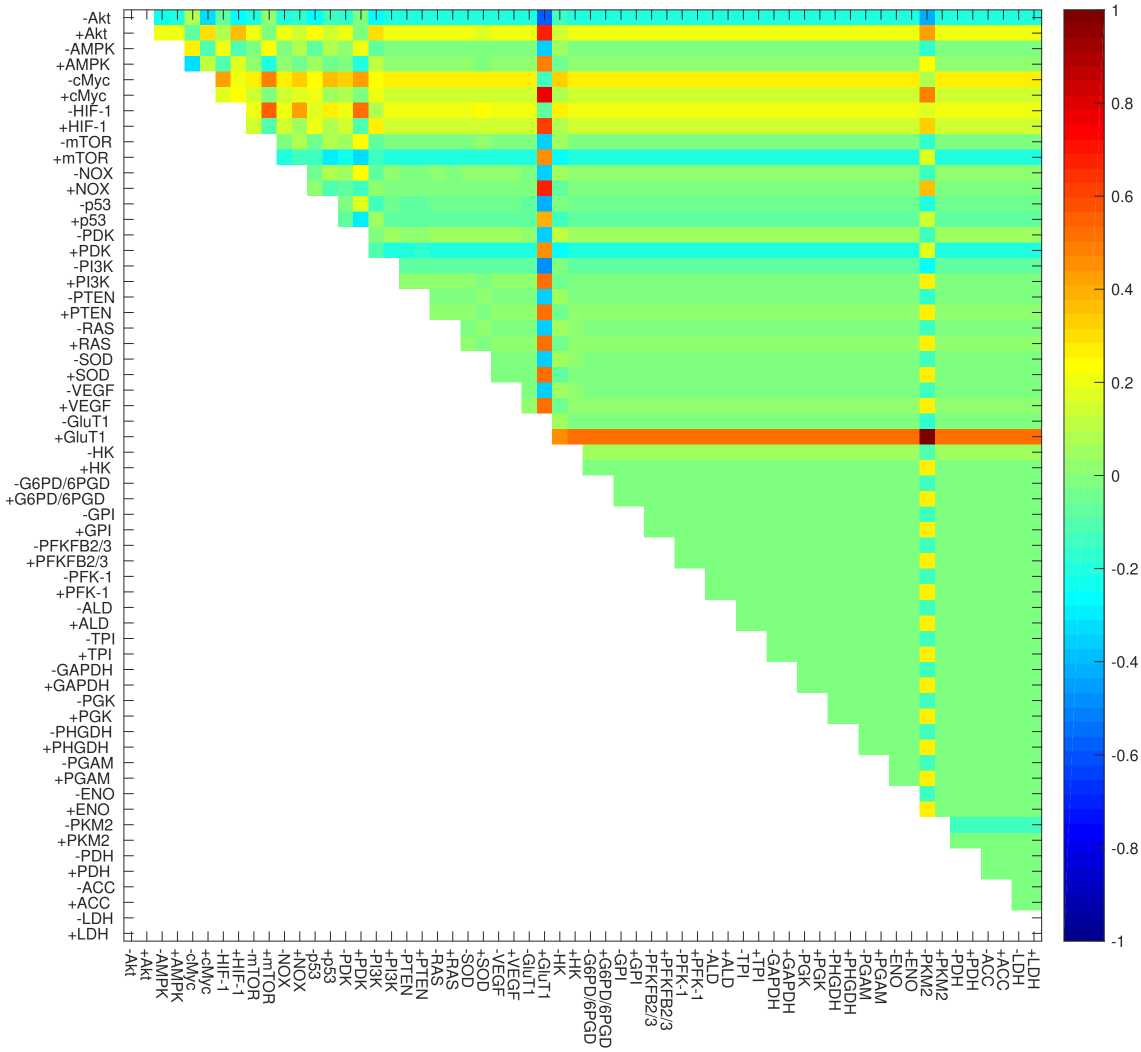


Figure S8: Predictions of combined metabolic therapeutic target for OXPHOS cancer based on barrier changes (related to Figure 10). Red color represents positive therapy and blue color represents negative therapy. The parameter $c_i$ = 1e-4.

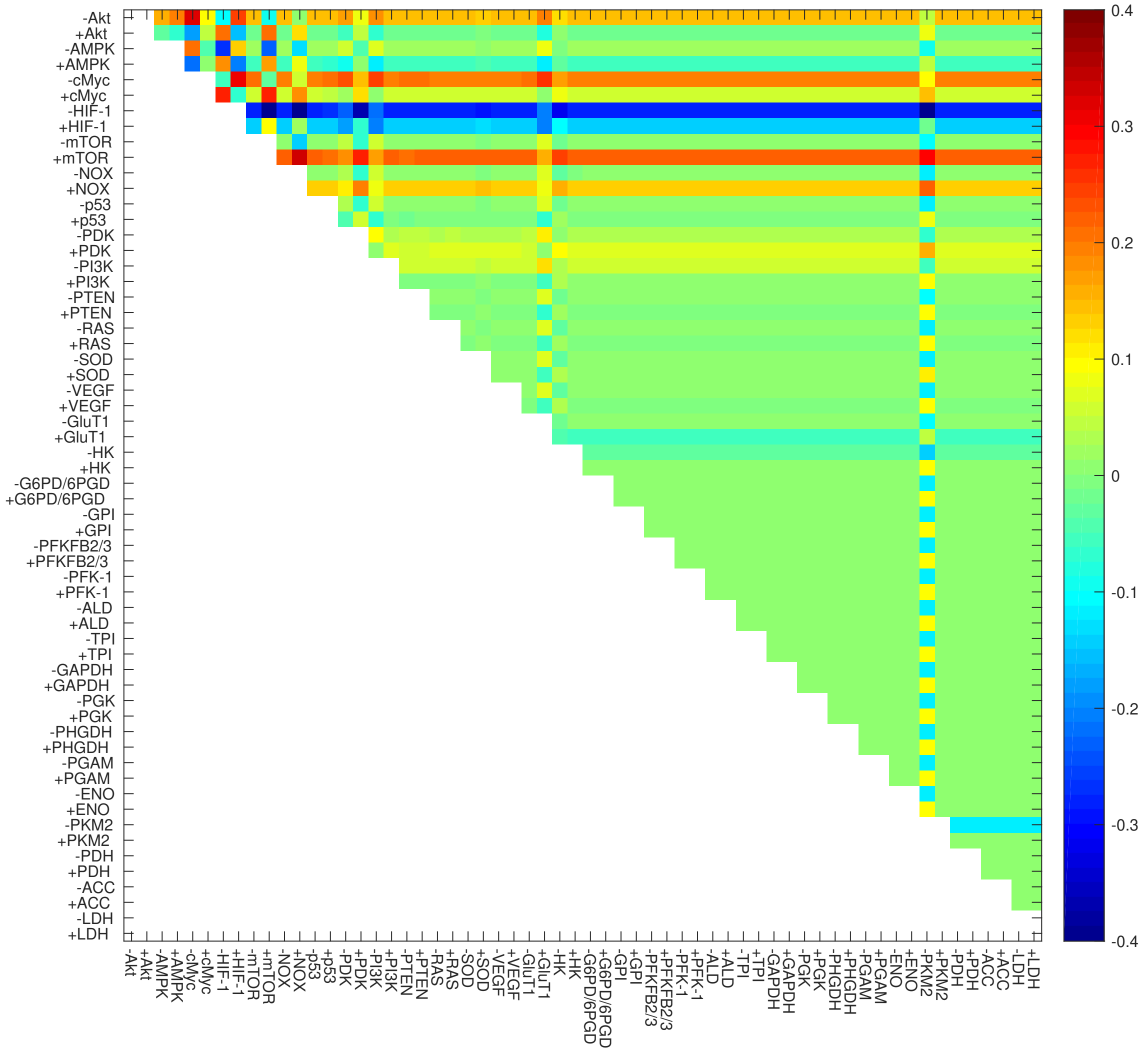


Figure S9: Predictions of combined metabolic therapeutic target for glycolysis cancer based on barrier changes (related to Figure 10). Red color represents positive therapy and blue color represents negative therapy. The parameter $c_i$ = 1e-4.

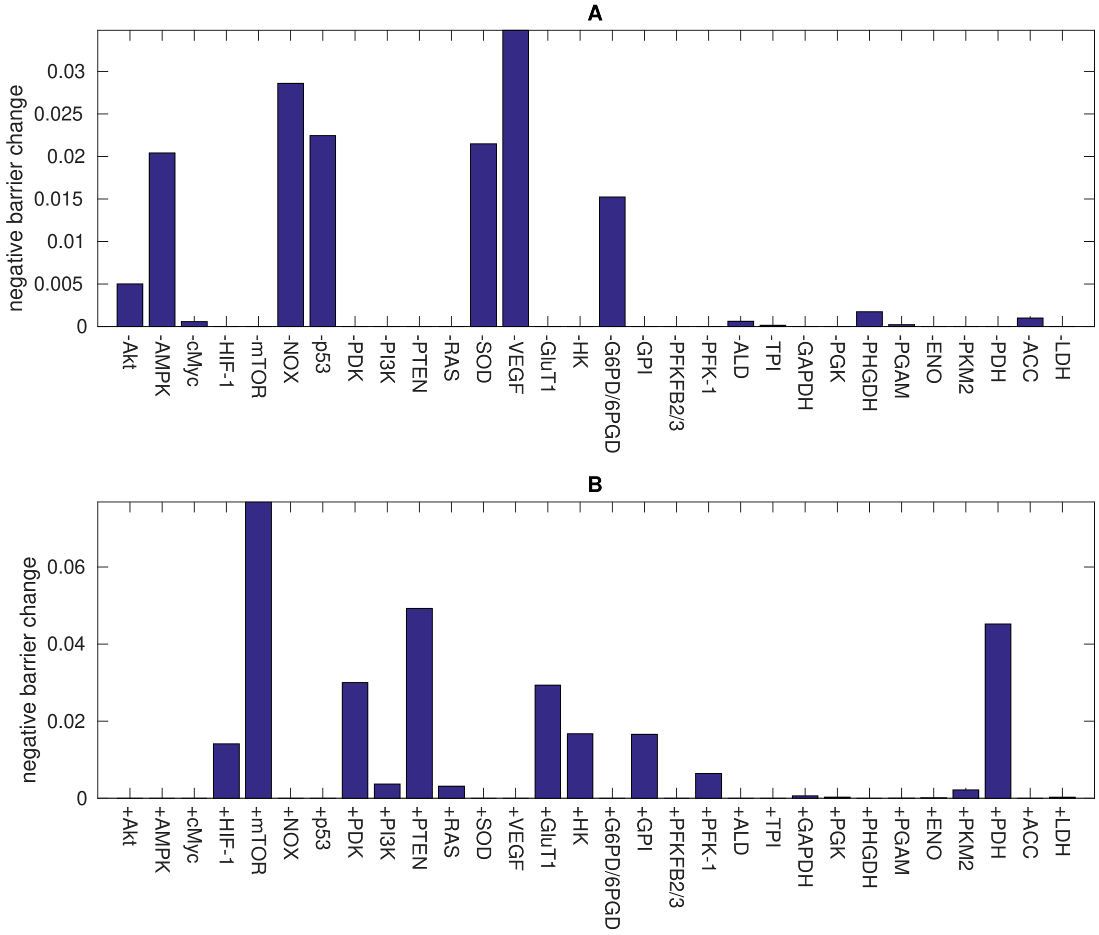


Figure S10: Predictions of metabolic target for cancer metabolism oscillation (related to Figure 10). The parameter $c_i$ = 1e-4. (A)Therapeutic effect for inhibiting the expressions of the genes and the enzymes. (B)Therapeutic effect for promoting the expressions of the genes and the enzymes.

Table S1: Genes, enzymes and metabolites for cancer gene-metabolism integrative network modeling (related to Figure 1).

| Genes | Enzymes | | Metabolites | |
|---|---|---|---|---|
| Gene symbol | Abbr. | Name | Abbr. | Name |
| Akt | GluT1 | Glucose transporter 1 | Glu | Glucose |
| AMPK | HK | Hexokinase | G6P | Glucose 6-phosphate |
| cMyc | G6PD/6PGD | glucose-6-phosphate dehydrogenase/Phosphogluconate dehydrogenase | F6P | Fructose 6-phosphate |
| HIF-1 | GPI | Phosphoglucose isomerase | FBP | Fructose 1,6-bisphosphate |
| mTOR | PFKFB2/3 | 6-phosphofructo-2-kinase/fructose-2,6-biphosphatase 2/3 | G3P | Glyceraldehyde 3-phosphate |
| NOX | PFK-1 | Phosphofructokinase | DHAP | Dihydroxyacetone phosphate |
| p53 | ALD | Aldolase | 1,3BPG | 1,3-Bisphosphoglycerate |
| PDK | TPI | Triose phosphate isomerase | 3PG | 3-phosphoglycerate |
| PI3K | GAPDH | Glyceraldehyde 3-phosphate dehydrogenase | 2PG | 2-phosphoglycerate |
| PTEN | PGK | Phosphoglycerate kinase | PEP | phosphoenolpyruvate |
| RAS | PHGDH | Phosphoglycerate dehydrogenase | Pyr | Pyruvate |
| SOD | PGAM | Phosphoglycerate mutase | Lac | Lactate |
| VEGF | ENO | Enolase | R5P | Ribose 5-phosphate |
| | PKM2 | Pyruvate kinase isozymes M2 | F2,6BP | Fructose 2,6-bisphosphate |
| | PDH | Pyruvate dehydrogenase | Ser | Serine |
| | ACC | Acetyl-CoA carboxylase | Cit | Citrate |
| | LDH | Lactate dehydrogenase | AMP | Adenosine monophosphate |
| | | | ADP | Adenosine diphosphate |
| | | | ATP | Adenosine triphosphate |
| | | | $NAD^+$ | Nicotinamide adenine dinucleotide |
| | | | NADH | Nicotinamide adenine dinucleotide reduced |
| | | | complex2 | Succinate-Q reductase |
| | | | ROS | Reactive oxygen species |

Table S2: Regulations among genes, enzymes and metabolites (related to Figure 1).

| Source | Target | p/n | type | Refs |
|---|---|---|---|---|
| Akt | cMyc | p | g2g | (Landeghem et al., 2013; Lien et al., 2016) |
| Akt | p53 | n | g2g | (Landeghem et al., 2013) |
| Akt | mTOR | p | g2g | (Lien et al., 2016) |
| AMPK | Akt | p | g2g | (Landeghem et al., 2013) |
| AMPK | mTOR | n | g2g | (Mulukutla et al., 2010) |
| AMPK | NOX | n | g2g | (Song and Zou, 2012; Wang et al., 2010c) |
| cMyc | HIF-1 | p | g2g | (Faubert et al., 2013; Doe et al., 2011) |
| cMyc | VEGF | p | g2g | (Landeghem et al., 2013) |
| HIF-1 | AMPK | n | g2g | (Emerling et al., 2009; Zhang et al., 2008) |
| HIF-1 | NOX | p | g2g | (Yuan et al., 2011) |
| HIF-1 | PDK | p | g2g | (Justus et al., 2015; Courtnay et al., 2015; Lien et al., 2016) |
| HIF-1 | VEGF | p | g2g | (Justus et al., 2015; Courtnay et al., 2015) |
| mTOR | HIF-1 | p | g2g | (Lien et al., 2016; Harada et al., 2008) |
| p53 | cMyc | n | g2g | (Landeghem et al., 2013) |
| p53 | HIF-1 | n | g2g | (Landeghem et al., 2013) |
| p53 | PDK | n | g2g | (Saunier et al., 2015) |
| p53 | PTEN | p | g2g | (Landeghem et al., 2013) |
| PDK | Akt | p | g2g | (Landeghem et al., 2013) |
| PI3K | Akt | p | g2g | (Courtnay et al., 2015) |
| PI3K | mTOR | p | g2g | (Courtnay et al., 2015) |
| PI3K | VEGF | p | g2g | (Landeghem et al., 2013) |
| PTEN | HIF-1 | n | g2g | (Landeghem et al., 2013) |
| PTEN | p53 | p | g2g | (Landeghem et al., 2013) |
| PTEN | PI3K | n | g2g | (Landeghem et al., 2013; Courtnay et al., 2015) |
| RAS | AMPK | p | g2g | (Mihaylova and Shaw, 2011) |
| RAS | HIF-1 | p | g2g | (Mihaylova and Shaw, 2011; Lim et al., 2004) |
| RAS | NOX | p | g2g | (Landeghem et al., 2013) |
| RAS | PI3K | p | g2g | (Landeghem et al., 2013) |
| SOD | p53 | p | g2g | (Landeghem et al., 2013) |
| VEGF | AMPK | p | g2g | (Landeghem et al., 2013) |
| VEGF | RAS | n | g2g | (Landeghem et al., 2013) |
| VEGF | SOD | p | g2g | (Landeghem et al., 2013) |
| ATP | AMPK | n | m2g | (Wegner et al., 2015) |
| ATP | PDK | p | m2g | (Saunier et al., 2015) |
| Lactate | HIF-1 | p | m2g | (Pavlova and Thompson, 2016) |
| Lactate | PI3K | p | m2g | (Pavlova and Thompson, 2016) |
| Lactate | VEGF | p | m2g | (Pavlova and Thompson, 2016) |
| R5P | AMPK | n | m2g | (Hammad et al., 2016) |
| ROS | cMyc | p | m2g | (Landeghem et al., 2013) |
| ROS | HIF-1 | p | m2g | (Li et al., 2014; Brunelle et al., 2005) |
| ROS | NOX | p | m2g | (Landeghem et al., 2013) |
| ROS | PI3K | p | m2g | (Landeghem et al., 2013) |
| ROS | RAS | p | m2g | (Landeghem et al., 2013) |
| ROS | SOD | p | m2g | (Landeghem et al., 2013) |
| ROS | VEGF | p | m2g | (Landeghem et al., 2013) |
| G6P | ACC | p | m2e | (Wegner et al., 2015) |
| F2,6BP | ACC | p | m2e | (Wegner et al., 2015) |
| Akt | GluT1 | p | g2e | (Courtnay et al., 2015; Lien et al., 2016) |
| Akt | HK | p | g2e | (Courtnay et al., 2015; Lien et al., 2016) |
| Akt | PFKFB2/3 | p | g2e | (Lien et al., 2016) |
| AMPK | ACC | p | g2e | (Wegner et al., 2015) |
| AMPK | PFKFB2/3 | p | g2e | (Landeghem et al., 2013) |
| cMyc | GluT1 | p | g2e | (Justus et al., 2015) |
| cMyc | LDH | p | g2e | (Landeghem et al., 2013; Justus et al., 2015; Lien et al., 2016) |
| HIF-1 | GluT1 | p | g2e | (Justus et al., 2015; Courtnay et al., 2015) |

| | | | | |
|---|---|---|---|---|
| HIF-1 | HK | p | g2e | (Landeghem et al., 2013; Justus et al., 2015; Courtnay et al., 2015) |
| HIF-1 | G6PD/6PGD | p | g2e | (Landeghem et al., 2013) |
| HIF-1 | GPI | p | g2e | (Landeghem et al., 2013) |
| HIF-1 | PFKFB2/3 | p | g2e | (Landeghem et al., 2013) |
| HIF-1 | PFK-1 | p | g2e | (Hasawi et al., 2014) |
| HIF-1 | ALD | p | g2e | (Landeghem et al., 2013) |
| HIF-1 | TPI | p | g2e | (Landeghem et al., 2013) |
| HIF-1 | GAPDH | p | g2e | (Landeghem et al., 2013) |
| HIF-1 | PGK | p | g2e | (Landeghem et al., 2013) |
| HIF-1 | PGAM | p | g2e | (Landeghem et al., 2013) |
| HIF-1 | ENO | p | g2e | (Landeghem et al., 2013) |
| HIF-1 | PKM2 | p | g2e | (Landeghem et al., 2013; Justus et al., 2015) |
| HIF-1 | LDH | p | g2e | (Justus et al., 2015; Courtnay et al., 2015) |
| p53 | GluT1 | n | g2e | (Justus et al., 2015) |
| p53 | G6PD/6PGD | p | g2e | (Justus et al., 2015) |
| PDK | PDH | n | g2e | (Justus et al., 2015; Lien et al., 2016) |
| PTEN | PDH | n | g2e | (Landeghem et al., 2013) |
| RAS | PFK-1 | p | g2e | (Hasawi et al., 2014) |

There is three types of regulations, g2g, m2g and g2e. The g2g type represents the regulations between genes. The m2g type represents the regulations from metabolite to gene. The g2e type represents the regulations from gene to enzyme.

Table S3: Metabolic reactions of glycolysis, TCA and oxidative phosphorylation (related to Figure 1).

| | Enzymes | Reactions |
|---|---|---|
| r1 | GluT1 | $Glu_{out} \rightleftharpoons Glu_{in}$ |
| r2 | HK | $Glu_{in}$ + ATP $\rightleftharpoons$ G6P + ADP |
| r3 | GPI | G6P $\rightleftharpoons$ F6P |
| r4 | PFK-1 | F6P + ATP $\rightleftharpoons$ FBP + ADP |
| r5 | ALD | FBP $\rightleftharpoons$ DHAP + G3P |
| r6 | TPI | DHAP $\rightleftharpoons$ G3P |
| r7 | GAPDH | G3P + $NAD^+$ $\rightleftharpoons$ 1,3BPG + NADH |
| r8 | PGK | 1,3BPG + ADP $\rightleftharpoons$ 3PG + ATP |
| r9 | PGAM | 3PG $\rightleftharpoons$ 2PG |
| r10 | ENO | 2PG $\rightleftharpoons$ PEP |
| r11 | PKM2 | PEP + ADP $\rightleftharpoons$ Pyruvate + ATP |
| r12 | LDH | Pyruvate + NADH $\rightleftharpoons$ Lactate + $NAD^+$ |
| r13 | G6PD_6PGD | G6P $\rightleftharpoons$ R5P |
| r14 | ATPases | ATP $\longrightarrow$ ADP |
| r15 | AK | AMP + ATP $\rightleftharpoons$ 2ADP |
| r16 | PFKFB2_3 | F6P $\rightleftharpoons$ F2,6BP |
| r17 | PHGDH | 3PG $\longrightarrow$ Serine |
| r18 | PDH | Pyruvate + ADP $\longrightarrow$ Citrate + ATP + Complex2 |
| r19 | ACC | Complex2 + 3ATP + AC-CoA $\longrightarrow$ mal-CoA + 3ADP + $NAD^+$ |
| r20 | SOD | ROS $\longrightarrow$ Null |
| r21 | | Lactate $\longrightarrow$ Null |
| r22 | | 3R5P $\rightleftharpoons$ 2F6P + G3P |
| r23 | NUCLEOTIDE BIOSYNTHESIS | R5P $\longrightarrow$ Null |
| r24 | SERINE CONSUMPTION | Serine $\longrightarrow$ Null |
| r25 | GPDH | NADH + ADP $\longrightarrow$ Complex2 + ATP + $NAD^+$ |
| r26 | | Citrate + 3ADP $\longrightarrow$ 3ATP + 4Complex2 |
| r27 | | Complex2 + 1.5ADP $\longrightarrow$ 1.5ATP |
| r28 | | Complex2 $\longrightarrow$ ROS |
| r29 | NOX | null $\longrightarrow$ ROS |
| r30 | | Citrate $\longrightarrow$ Null |

Table S4: Parameters for modeling the regulations (related to Figure 1).

| Parameter | Value | Parameter | Value | Parameter | Value |
|---|---|---|---|---|---|
| $\gamma_{Akt->cMyc}$ | 2.23 | $\gamma_{Akt->mTOR}$ | 1.33 | $\gamma_{Akt->p53}$ | 0.1 |
| $\gamma_{Akt->GluT1}$ | 1.5 | $\gamma_{Akt->HK}$ | 1.5 | $\gamma_{Akt->PFKFB2/3}$ | 1.71 |
| $\gamma_{AMPK->Akt}$ | 2.26 | $\gamma_{AMPK->mTOR}$ | 0.21 | $\gamma_{AMPK->NOX}$ | 0.72 |
| $\gamma_{AMPK->PFKFB2/3}$ | 1.84 | $\gamma_{AMPK->ACC}$ | 1.51 | $\gamma_{cMyc->HIF-1}$ | 2 |
| $\gamma_{cMyc->VEGF}$ | 3.78 | $\gamma_{cMyc->GluT1}$ | 1.58 | $\gamma_{cMyc->LDH}$ | 2.6 |
| $\gamma_{HIF-1->AMPK}$ | 0.08 | $\gamma_{HIF-1->NOX}$ | 2.82 | $\gamma_{HIF-1->PDK}$ | 5.81 |
| $\gamma_{HIF-1->VEGF}$ | 3.77 | $\gamma_{HIF-1->GluT1}$ | 2.41 | $\gamma_{HIF-1->HK}$ | 1.57 |
| $\gamma_{HIF-1->G6PD/6PGD}$ | 1.12 | $\gamma_{HIF-1->GPI}$ | 1.01 | $\gamma_{HIF-1->PFKFB2/3}$ | 1 |
| $\gamma_{HIF-1->PFK-1}$ | 1.78 | $\gamma_{HIF-1->ALD}$ | 1.03 | $\gamma_{HIF-1->TPI}$ | 1.46 |
| $\gamma_{HIF-1->GAPDH}$ | 3.07 | $\gamma_{HIF-1->PGK}$ | 3.54 | $\gamma_{HIF-1->PGAM}$ | 2.52 |
| $\gamma_{HIF-1->ENO}$ | 1.28 | $\gamma_{HIF-1->PKM2}$ | 2.18 | $\gamma_{HIF-1->LDH}$ | 3.61 |
| $\gamma_{mTOR->HIF-1}$ | 3 | $\gamma_{p53->cMyc}$ | 0.29 | $\gamma_{p53->HIF-1}$ | 0.4 |
| $\gamma_{p53->PDK}$ | 0.8 | $\gamma_{p53->PTEN}$ | 10 | $\gamma_{p53->GluT1}$ | 0.8 |
| $\gamma_{p53->G6PD/6PGD}$ | 1.38 | $\gamma_{PDK->Akt}$ | 5.69 | $\gamma_{PDK->PDH}$ | 0.14 |
| $\gamma_{PI3K->Akt}$ | 2 | $\gamma_{PI3K->mTOR}$ | 1.08 | $\gamma_{PI3K->VEGF}$ | 1.25 |
| $\gamma_{PTEN->HIF-1}$ | 0.36 | $\gamma_{PTEN->p53}$ | 5.86 | $\gamma_{PTEN->PI3K}$ | 0.83 |
| $\gamma_{PTEN->PDH}$ | 0.09 | $\gamma_{RAS->AMPK}$ | 3.47 | $\gamma_{RAS->HIF-1}$ | 1.5 |
| $\gamma_{RAS->NOX}$ | 7.39 | $\gamma_{RAS->PI3K}$ | 28.78 | $\gamma_{RAS->PFK-1}$ | 1.41 |
| $\gamma_{SOD->p53}$ | 12.87 | $\gamma_{VEGF->AMPK}$ | 8.36 | $\gamma_{VEGF->RAS}$ | 0.06 |
| $\gamma_{VEGF->SOD}$ | 2 | $\gamma_{G6P->ACC}$ | 1.21 | $\gamma_{Lactate->HIF-1}$ | 10.94 |
| $\gamma_{Lactate->PI3K}$ | 4.22 | $\gamma_{Lactate->VEGF}$ | 3 | $\gamma_{R5P->AMPK}$ | 0.05 |
| $\gamma_{F2,6BP->ACC}$ | 1.34 | $\gamma_{ATP->AMPK}$ | 0.36 | $\gamma_{ATP->PDK}$ | 29.6 |
| $\gamma_{ROS->cMyc}$ | 4.78 | $\gamma_{ROS->HIF-1}$ | 10 | $\gamma_{ROS->NOX}$ | 4.07 |
| $\gamma_{ROS->PI3K}$ | 5.12 | $\gamma_{ROS->RAS}$ | 5.99 | $\gamma_{ROS->SOD}$ | 2.03 |
| $\gamma_{ROS->RAS}$ | 3 | A | 0.005 | D | 0.005 |

Table S5: Parameters for modeling the metabolic reactions (related to Figure 1).

| Parameter | Value | Refs | Parameter | Value | Refs |
|---|---|---|---|---|---|
| Gluout | 5 | (Marín-Hernández et al., 2011) | Pi | 4 | (Marín-Hernández et al., 2011) |
| bx6PG | 0.39 | (Marín-Hernández et al., 2011) | Ery4P | 0.016 | (Marín-Hernández et al., 2011) |
| Lacout | 2.57 | (Marín-Hernández et al., 2014) | O2 | 0.03 | |
| Citrate | 1.7 | (Moreno-Sánchez et al., 2010) | Vmf_1 | 0.03 | (Marín-Hernández et al., 2011) |
| Keq_1 | 1 | (Marín-Hernández et al., 2011) | Kgluout_1 | 9.3 | (Rodríguez-Enríquez et al., 2009) |
| Kgluin_1 | 10 | (Marín-Hernández et al., 2011) | Vm_2 | 0.0475 | (Marín-Hernández et al., 2011) |
| Ka_2 | 0.1 | (Marín-Hernández et al., 2011) | Kb_2 | 1.1 | (Marín-Hernández et al., 2011) |
| Keq_2 | 651 | (Marín-Hernández et al., 2011) | Kp_2 | 0.02 | (Wilson, 2003) |
| Kq_2 | 3.5 | (Marín-Hernández et al., 2011) | Vmf_3 | 0.24 | |
| Kg6p_3 | 0.4 | (Marín-Hernández et al., 2014) | Vmr_3 | 0.54 | |
| Kf6p_3 | 0.05 | (Marín-Hernández et al., 2014) | Kery4p_3 | 0.001 | (Marín-Hernández et al., 2014) |
| Kfbp_3 | 0.06 | (Marín-Hernández et al., 2014) | Kpg_3 | 0.015 | (Marín-Hernández et al., 2014) |
| Vm_4 | 0.026 | (Marín-Hernández et al., 2014) | Katp_4 | 0.0292 | (Marín-Hernández et al., 2014) |
| beta_4 | 1.18 | (Marín-Hernández et al., 2014) | alfa_4 | 0.75 | (Marín-Hernández et al., 2014) |
| Kf26bp_4 | 0.00099 | (Marín-Hernández et al., 2014) | Kf6p_4 | 1.1 | (Marín-Hernández et al., 2014) |
| L_4 | 6.6 | (Marín-Hernández et al., 2014) | Kcit_4 | 6.7 | (Marín-Hernández et al., 2014) |
| Kiatp_4 | 1.1 | (Marín-Hernández et al., 2014) | Kadp_4 | 5 | (Marín-Hernández et al., 2011) |
| Kfbp_4 | 5 | (Marín-Hernández et al., 2011) | Keq_4 | 247 | (Marín-Hernández et al., 2014) |
| Vmf_5 | 0.08 | (Marín-Hernández et al., 2011) | Kfbp_5 | 0.009 | (Marín-Hernández et al., 2011) |
| Vmr_5 | 0.063 | (Marín-Hernández et al., 2011) | Kdhap_5 | 0.08 | (Marín-Hernández et al., 2011) |
| Kg3p_5 | 0.16 | (Marín-Hernández et al., 2011) | Kms_6 | 1.6 | (Marín-Hernández et al., 2011) |
| Kmp_6 | 0.51 | (Marín-Hernández et al., 2011) | Vf_6 | 3.4 | (Marín-Hernández et al., 2011) |
| Vr_6 | 28 | (Marín-Hernández et al., 2011) | Vmf_7 | 0.58 | (Marín-Hernández et al., 2011) |
| Knad_7 | 0.09 | (Marín-Hernández et al., 2011) | Kg3p_7 | 0.19 | (Marín-Hernández et al., 2011) |
| Kp_7 | 29 | (Marín-Hernández et al., 2011) | Vmr_7 | 0.72 | (Marín-Hernández et al., 2011) |
| Kdpg_7 | 0.022 | (Marín-Hernández et al., 2011) | Knadh_7 | 0.01 | (Marín-Hernández et al., 2011) |
| Vmf_8 | 8.7 | (Marín-Hernández et al., 2011) | alfa_8 | 1 | (Marín-Hernández et al., 2011) |
| Ka_8 | 0.079 | (Marín-Hernández et al., 2011) | Kb_8 | 0.04 | (Marín-Hernández et al., 2011) |
| Vmr_8 | 2.5 | (Marín-Hernández et al., 2011) | beta_8 | 1 | (Marín-Hernández et al., 2011) |
| Kp_8 | 0.13 | (Marín-Hernández et al., 2011) | Kq_8 | 0.27 | (Marín-Hernández et al., 2011) |
| Kms_9 | 0.19 | (Marín-Hernández et al., 2011) | Kmp_9 | 0.12 | (Marín-Hernández et al., 2011) |
| Vf_9 | 0.94 | (Marín-Hernández et al., 2011) | Vr_9 | 0.36 | (Marín-Hernández et al., 2011) |
| Kms_10 | 0.038 | (Marín-Hernández et al., 2011) | Kmp_10 | 0.06 | (Marín-Hernández et al., 2011) |
| Vf_10 | 0.34 | (Marín-Hernández et al., 2011) | Vr_10 | 0.38 | (Marín-Hernández et al., 2011) |
| Vmax_11 | 0.083333 | (Marín-Hernández et al., 2014) | Kpep_11 | 0.05 | (Marín-Hernández et al., 2014) |
| Kadp_11 | 0.4 | (Marín-Hernández et al., 2014) | Keq_11 | 195172.4 | (Marín-Hernández et al., 2014) |
| Kpyr_11 | 10 | (Marín-Hernández et al., 2014) | Katp_11 | 0.86 | (Marín-Hernández et al., 2014) |
| Vmf_12 | 3.4 | (Marín-Hernández et al., 2011) | alfa_12 | 1 | (Marín-Hernández et al., 2011) |
| Ka_12 | 0.002 | (Marín-Hernández et al., 2011) | Kb_12 | 0.3 | (Marín-Hernández et al., 2011) |
| Vmr_12 | 0.54 | (Marín-Hernández et al., 2011) | beta_12 | 1 | (Marín-Hernández et al., 2011) |
| Kp_12 | 4.7 | (Marín-Hernández et al., 2011) | Kq_12 | 0.07 | (Marín-Hernández et al., 2011) |
| Vm_13 | 0.01 | | Km_13 | 0.5 | |
| k_14 | 0.01 | | k1_15 | 1 | (Marín-Hernández et al., 2011) |
| k2_15 | 2.26 | (Marín-Hernández et al., 2011) | Vf_16 | 0.5 | |
| Vr_16 | 0.1 | | Kms_16 | 0.5 | |
| Kmp_16 | 0.5 | | Vm_17 | 0.001 | |
| Km_17 | 0.5 | | Vm_18 | 0.2 | |
| Km_18 | 0.5 | | Vm_19 | 0.01 | |
| Km_19 | 0.5 | | Vm_20 | 0.01 | |
| Km_20 | 0.05 | | Vmf_21 | 0.053333 | (Marín-Hernández et al., 2014) |
| Keq_21 | 1 | (Marín-Hernández et al., 2014) | Klacin_21 | 8.5 | (Marín-Hernández et al., 2014) |
| Klacout_21 | 0.5 | (Marín-Hernández et al., 2014) | k1_22 | 0.001 | |
| k2_22 | 0.01 | | k_23 | 0.01 | |
| k_24 | 0.001 | | k_25 | 0.074 | |
| k_26 | 0.015 | | k_27 | 0.1 | |

| | | | | | |
|---|---|---|---|---|---|
| k_28 | 0.5 | | Vm_29 | 0.05 | |
| Km_29 | 0.5 | | k_30 | 0.01 | |

Table S6: Genes related to glycolysis, TCA cycle and oxidative phosphorylation (related to Figure 2).

| Glycolysis | |
|---|---|
| Gene/Enzyme | Symbols |
| Akt | AKT1<br>AKT2<br>AKT3 |
| HIF-1 | HIF1A<br>HIF1B |
| mTOR<br>NOX | MTOR<br>NOX1<br>NOX2<br>NOX3<br>NOX4<br>NOX5<br>DUOX1<br>DUOX2 |
| PDK | PDK1<br>PDK2<br>PDK3<br>PDK4 |
| PI3K | PIK3CA<br>PIK3CB<br>PIK3CG<br>PIK3CD<br>PIK3R1<br>PIK3R2<br>PIK3R3<br>PIK3R4<br>PIK3R5<br>PIK3R6<br>PIK3C2A<br>PIK3C2B<br>PIK3C2G<br>PIK3C3 |
| RAS | KRAS<br>HRAS<br>NRAS |
| Hexokinase | HK1<br>HK2<br>HK3<br>HKDC1 |
| Phosphoglucose isomerase | GPI |
| Phosphofructokinase | PFKL<br>PFKM<br>PFKP |
| Aldolase | ALDOA<br>ALDOB<br>ALDOC |
| Triose phosphate isomerase | TPI1 |
| Glyceraldehyde 3-phosphate dehydrogenase | GAPDH<br>GAPDHS |
| Phosphoglycerate kinase | PGK1<br>PGK2 |
| Phosphoglycerate mutase | PGAM4<br>PGAM1<br>PGAM2<br>BPGM |
| Enolase | ENO1<br>ENO2 |

| TCA cycle/OXPHOS | |
|---|---|
| Gene/Enzyme | Symbols |
| AMPK | PRKAA1<br>PRKAA2<br>PRKAB1<br>PRKAB2<br>PRKAG1<br>PRKAG2<br>PRKAG3 |
| cMyc | MYC |
| p53 | TP53 |
| SOD | SOD1<br>SOD2<br>SOD3 |
| Pyruvate dehydrogenase complex | PDHA1<br>PDHA2<br>PDHB<br>DLAT<br>DLD |
| Citrate synthase | CS |
| Aconitase | ACO1<br>ACO2 |
| Isocitrate dehydrogenase | IDH3A<br>IDH3B<br>IDH3G<br>IDH1<br>IDH2 |
| Alpha-Ketoglutarate dehydrogenase complex | OGDH<br>DHTKD1<br>OGDHL<br>DLST |
| Succinyl CoA synthetase | SUCLG2<br>SUCLG1<br>SUCLA2 |
| Succinate dehydrogenase | SDHA<br>SDHB |
| Fumarase | FH |
| Malate dehydrogenase | MDH1<br>MDH2 |
| NADH-Q oxidoreductase | NDUFS7<br>ND1<br>ND2<br>ND3<br>ND4<br>ND4L<br>ND5<br>ND6<br>NDUFS1<br>NDUFS2<br>NDUFS3<br>NDUFV1<br>NDUFS8<br>NDUFV2 |
| Succinate-Q oxidoreductase | SDHC<br>SDHD |
| Q-cytochrome c oxidoreductase | UQCRFS1 |
| Cytochrome c oxiddase | COX1 |

| | | | |
|---|---|---|---|
| | ENO3<br>ENO4 | | |
| Pyruvate kinase | PKLR<br>PKM | | |
| lactate dehydrogenase | LDHA<br>LDHB | | |

# Transparent Methods

## Metabolic Kinetic Equations

The metabolic reactions are listed in Table S3. The metabolic reactions contain single directional and bidirectional reactions. For every bidirectional reaction, which is reversible reaction, the kinetic velocity can be positive or negative determined by the metabolite concentrations. As an example, the simplest representation of enzyme-catalyzed reversible reaction is(Segel, 1975):

$$\mathrm{E + S} \underset{k_{-1}}{\overset{k_1}{\rightleftharpoons}} \mathrm{ES} \underset{k_{-2}}{\overset{k_2}{\rightleftharpoons}} \mathrm{E + P}$$

Each k represents rate constant for each reaction respectively. If we focus on only one central complex , the net velocity in the forward direction is given by:

$$v_{net} = k_2[ES] - k_{-2}[E][P] \tag{1}$$

The condition at steady state is given as:

$$\frac{d[ES]}{dt} = 0 \tag{2}$$

$$+\frac{d[ES]}{dt} = k_1[E][S] + k_{-2}[E][P] \tag{3}$$

$$-\frac{d[ES]}{dt} = k_2[ES] + k_{-1}[ES] \tag{4}$$

Eq. 1 can be transformed into(Segel, 1975):

$$v_{net} = \frac{V_{maxf}\frac{[S]}{K_{ms}} - V_{maxr}\frac{[P]}{K_{mp}}}{1 + \frac{[S]}{K_{ms}} + \frac{[P]}{K_{mp}}} \tag{5}$$

where:

$$k_2[E]_t = V_{maxf} \tag{6}$$

$$k_{-1}[E]_t = V_{maxr} \tag{7}$$

$$\frac{k_2 + k_{-1}}{k_1} = K_{ms} \tag{8}$$

$$\frac{k_2 + k_{-1}}{k_{-2}} = K_{mp} \tag{9}$$

$$[E]_t = [E] + [ES] \tag{10}$$

[ES] represents the concentration of total enzyme. $V_{maxf}$ represents the maximal velocity in the forward reaction and $V_{maxr}$ represents the maximal velocity in the reverse reaction.

The metabolic kinetic velocities are showed as follow.

1. Reaction r1(GluT1):

$$v = Vmf \frac{[Glu_{out}] - [Glu_{in}]/Keq}{K_{Gluout}(1 + [Glu_{in}]/K_{Gluin}) + Glu_{out}} \tag{11}$$

The kinetics of GluT1 is described as a monosubstrate reversible Michaelis–Menten equation (Segel, 1975). $Glu_{out}$ and $Glu_{in}$ are the extra-cellular and intra-cellular glucose concentrations. $K_{Gluout}$ and $K_{Gluin}$ are the enzyme's affinity respectively. $Keq$ is the equilibrium constant. $Vmf$ is the maximal velocity in the forward reaction.

2. Reaction r2(HK) and reaction r11(PKM2):

$$v = Vmf \frac{([A][B] - \frac{[P][Q]}{Keq})/(KaKb)}{1 + \frac{[A]}{Ka} + \frac{[B]}{Kb} + \frac{[A][B]}{KaKb} + \frac{[P]}{Kp} + \frac{[Q]}{Kq} + \frac{[P][Q]}{KpKq} + \frac{[A][Q]}{KaKq} + \frac{[P][B]}{KpKb}} \tag{12}$$

The kinetics of HK and PKM2 are described as random bi-substrate Michaelis–Menten (Segel, 1975). For reaction r2 [A] and [B] represent the $\mathrm{Glu_{in}}$ and ATP concentrations, whereas [P] and [Q] represent the G6P and ADP concentrations. For reaction r11 [A] and [B] represent the PEP and ADP concentrations, whereas [P] and [Q] represent the Pyruvate and ATP concentration. Ka, Kb, Kp and Kq represent the enzymes Km values for their respective ligands.

3. Reaction r3(GPI):

$$v = \frac{Vmf\frac{[G6P]}{K_{G6P}} - Vmr\frac{[F6P]}{K_{F6P}}}{1 + \frac{[G6P]}{K_{G6P}} + \frac{[F6P]}{K_{F6P}} + \frac{[ERY4P]}{K_{ERY4P}} + \frac{[6PG]}{K_{6PG}} + \frac{[FBP]}{K_{FBP}}} \tag{13}$$

The kinetics of GPI is described as a monoreactant reversible equation with competitive inhibition by Ery4P, 6PG and FBP (Marín-Hernández et al., 2011). $Vmf$ is the maximal velocity in the forward reaction, while $Vmr$ is the maximal velocity in the reverse reaction.

4. Reaction r4(PFK-1):

$$v = Vm((\frac{\frac{[ATP]}{K_{ATP}}}{1 + \frac{[ATP]}{K_{ATP}}})(\frac{1 + \frac{\beta[F26BP]}{\alpha Ka_{F26BP}}}{1 + \frac{[F26BP]}{\alpha Ka_{F26BP}}})(\frac{\frac{[F6P](1+\frac{[F26BP]}{\alpha Ka_{F26BP}})}{K_{F6P}(1+\frac{[F26BP]}{Ka_{F26BP}}}))(1 + \frac{[F6P](1+\frac{[F26BP]}{\alpha Ka_{F26BP}})}{K_{F6P}(1+\frac{[F26BP]}{Ka_{F26BP}})})^3}{\frac{L(1+\frac{[Citrate]}{Ki_{Cit}})^4(1+\frac{[ATP]}{Ki_{ATP}})^4}{(1+\frac{[F26BP]}{Ka_{F26BP}})^4} + (1 + \frac{[F6P](1+\frac{[F26BP]}{\alpha Ka_{F26BP}})}{K_{F6P}(1+\frac{[F26BP]}{Ka_{F26BP}})})^4}) - \frac{\frac{[ADP][ATP]}{K_{ADP}K_{FBP}Keq}}{\frac{[ADP]}{K_{ADP}} + \frac{[FBP]}{K_{FBP}} + \frac{[ADP][FBP]}{K_{ADP}K_{FBP}} + 1}) \tag{14}$$

The kinetics of PFK-1 could be considered as the concerted transition model of Monod, Wyman and Changeux for exclusive ligand binding (F6P, activators, and inhibitors) together with mixed-type activation (F2,6BP or AMP or Pi) and simple Michaelis–Menten terms for ATP and reverse reaction (Marín-Hernández et al., 2011). L is the allosteric transition constant.

5. Reaction r5(ALD):

$$v = \frac{Vmf\frac{[FBP]}{K_{FBP}} - Vmr\frac{[DHAP][G3P]}{K_{DHAP}K_{G3P}}}{1 + \frac{[FBP]}{K_{FBP}} + \frac{[DHAP]}{K_{DHAP}} + \frac{[G3P]}{K_{G3P}} + \frac{[DHAP][G3P]}{K_{DHAP}K_{G3P}}} \tag{15}$$

The kinetics of ALD rate equation is the reversible Uni–Bi Michaelis–Menten equation(Segel, 1975). $Vmf$ is the maximal velocity in the forward reaction, while $Vmr$ is the maximal velocity in the reverse reaction.

6. Reaction r6(TPI), reaction r9(PGAM) and reaction r10(ENO):

$$v = \frac{Vmf\frac{[S]}{Ks} - Vmr\frac{[P]}{Kp}}{1 + \frac{[S]}{Ks} + \frac{[P]}{Kp}} \tag{16}$$

The kinetics of TPI, PGAM and ENO are described as monosubstrate simple reversible Michaelis–Menten equation (Segel, 1975). [S] and [P] represent the respective concentrations of substrates and products with their respective affinity constants. $Vmf$ is the maximal velocity in the forward reaction, while $Vmr$ is the maximal velocity in the reverse reaction.

7. Reaction r7(GAPDH):

$$v = \frac{Vmf\frac{[NAD][G3P][Pi]}{K_{NAD}K_{G3P}K_{Pi}} - Vmr\frac{[BPG][NADH]}{K_{BPG}K_{NADH}}}{1 + \frac{[NAD]}{K_{NAD}} + \frac{[NAD][G3P]}{K_{NAD}K_{G3P}} + \frac{[NAD][G3P][Pi]}{K_{NAD}K_{G3P}K_{Pi}} + \frac{[BPG][NADH]}{K_{DPG}K_{NADH}} + \frac{[NADH]}{K_{NADH}}} \tag{17}$$

The kinetics of GAPDH is described as a simplified ordered Ter–Bi reversible Michaelis–Menten equation (Marín-Hernández et al., 2011). $Vmf$ is the maximal velocity in the forward reaction, while $Vmr$ is the maximal velocity in the reverse reaction.

8. Reaction r8(PGK) and reaction r12(LDH):

$$v = \frac{Vmf\frac{[A][B]}{\alpha KaKb} - Vmr\frac{[P][Q]]}{\beta KpKq}}{1 + \frac{[A]}{Ka} + \frac{[B]}{Kb} + \frac{[A][B]}{\alpha KaKb} + \frac{[P][Q]}{\beta KpKq} + \frac{[P]}{Kp} + \frac{[Q]}{Kq}} \tag{18}$$

The kinetics of PGK and LDH are described as the random Bi–Bi reversible Michaelis–Menten equation(Marín-Hernández et al., 2011). For reaction r8, [A] and [B] represent the 1,3BPG and ADP concentrations, whereas [P] and [Q] represent the 3PG and ATP concentrations. For reaction r12, [A] and [B] represent the Pyruvate and NADH concentrations, whereas [P] and [Q] represent the intra-cellular Lactate and NAD concentrations. $Vmf$ is the maximal velocity in the forward reaction, while $Vmr$ is the maximal velocity in the reverse reaction.

9. Reaction r13(G6PD/6PGD), reaction r18(PDH), reaction r19(ACC) and reaction r20(SOD):

$$v = Vm\frac{[S]}{[S] + Km} \tag{19}$$

The kinetics of G6PD/6PGD, PDH, ACC, SOD are described as Michaelis–Menten equation. [S] represent the G6P, Pyruvate, complex2 and ROS concentrations respectively.

10. Reaction r14(ATPases):

$$v = k[ATP] \tag{20}$$

The kinetics of ATPases is described as the irreversible mass-action reaction, in which k is the rate constant.

11. Reaction r15(AK):

$$v = k1[ATP][AMP] - k2[ADP]^2; \tag{21}$$

The kinetics of AK is described as reversible mass-action reactions, in which k1 and k2 are the rate constants.

12. Reaction r16(PFKFB2/3):

$$v = \frac{Vmf\frac{[F6P]}{Ks} - Vmr\frac{[F2,6BP]}{Kp}}{1 + \frac{[F6P]}{Ks} + \frac{[F2,6BP]}{Kp}} \tag{22}$$

The kinetics of PFKFB2/3 is described as monosubstrate simple reversible Michaelis–Menten equation (Segel, 1975). $Vmf$ is the maximal velocity in the forward reaction, while $Vmr$ is the maximal velocity in the reverse reaction.

13. Reaction r17(PHGDH):

$$v = \frac{Vmf\frac{[3PG]}{Ks} - Vmr\frac{[Serine]}{Kp}}{1 + \frac{[3PG]}{Ks} + \frac{[Serine]}{Kp}} \tag{23}$$

The kinetics of PHGDH is described as monosubstrate simple reversible Michaelis–Menten equation (Segel, 1975). $Vmf$ is the maximal velocity in the forward reaction, while $Vmr$ is the maximal velocity in the reverse reaction.

14. Reaction r21:

$$v = \frac{Vmf([Lac_{in}] - \frac{[Lac_{out}]}{Keq})}{K_{lacin}(1 + \frac{[Lac_{out}]}{K_{lacout}}) + [Lac_{in}]} \tag{24}$$

The kinetics is described as the monosubstrate reversible Michaelis–Menten equation. $[\mathrm{Lac_{in}}]$ and $[\mathrm{Lac_{out}}]$ are the extra-cellular and intra-cellular lactate concentrations. $K_{lacin}$ and $K_{lacout}$ are the enzyme's affinity respectively. $Keq$ is the equilibrium constant.

15. Reaction r22:

$$v = k1[R5P]^3 - k2[F6P]^2[G3P]; \tag{25}$$

The kinetics is described as reversible mass-action reactions, in which k1 and k2 are the rate constants.

16. Reaction r23(NUCLEOTIDE BIOSYNTHESIS) and Reaction r24(SERINE CONSUMPTION):

$$v = k[S] \tag{26}$$

The kinetics of Nucleotide biosynthesis and Serine consumption are described as the irreversible mass-action reaction, in which k is the rate constant. [S] represent the R5P and Serine concentration respectively.

17. Reaction r25, Reaction r26 and Reaction r27:

$$v = k[S]; \tag{27}$$

The kinetics are described as the irreversible mass-action reaction, in which k is the rate constant. [S] represent the NADH, Citrate and complex2 concentration respectively.

18. Reaction r28:

$$v = k[Complex2][O_2]; \tag{28}$$

The kinetics is described as the irreversible mass-action reaction, in which k is the rate constant.

19. Reaction r29(NOX):

$$v = Vm\frac{[O_2]}{[O_2] + Km}; \tag{29}$$

The kinetics of NOX is described as Michaelis–Menten equation.

20. Reaction r30:

$$v = k[Citrate] \tag{30}$$

The kinetics is described as the irreversible mass-action reaction, in which k is the rate constant. Citrate consumption could be used for other biological functions such as substrate for fatty acids biosynthesis.

## Parameter Setting

The driving forces of the dynamics for the gene expression or enzyme levels regulated by one gene could be described as:

$$\dot{X} = AH(Y) - DX \tag{31}$$

$$H(Y) = \frac{S^n}{S^n + Y^n} + \gamma \frac{Y^n}{S^n + Y^n} \tag{32}$$

where X represents the regulated gene expression or enzyme level and Y represents expression of the regulating gene. At the steady state, where $\dot{X} = 0$, the regulated gene expression or enzyme levels can be determined as:

$$X = \frac{A}{D} H(Y) \tag{33}$$

As shown in Eq. 33, if there is no regulations on X($\gamma = 1$), the gene expression or enzyme level at steady state can be represented as:

$$X_0 = \frac{A}{D} \tag{34}$$

The fold change of gene expression regulated by gene Y can be represented as:

$$fold\ change_r = \frac{X}{X_0} = \frac{\frac{A}{D}H(Y)}{\frac{A}{D}} = H(Y) \tag{35}$$

The fold change of gene expression between cancer sample and normal sample can be represented as the ratio of gene expression level of cancer cell to normal cell at steady state:

$$fold\ change_{c2n} = \frac{X_c}{X_n} = \frac{H(Y_c)}{H(Y_n)} \tag{36}$$

$X_c$ and $X_n$ represent the gene expression level at steady state in cancer cell and normal cell respectively. If X is regulated by Y and $Y \to +\infty$, the gene expression or enzyme level is equal to $\gamma$(A/D). Thus the gene regulation strength $\gamma$ reflects the regulation of X from Y when saturate. We consider that the gene regulation could get the saturation in cancer cell($\gamma \approx H(Y_c)$)). The gene regulation strength can be represented as:

$$\gamma \approx H(Y_c) = H(Y_n) \times \frac{H(Y_c)}{H(Y_n)} = fold\ change_r \times fold\ change_{c2n} \tag{37}$$

The fold change of gene expressions in response to knockdown of certain gene is around 4 in adult fibroblasts(Trapnell et al., 2012). The fold changes of gene expressions between cancer and normal sample in all 13 cancer types reach 10 in previous studies(Hu et al., 2017). Thus we choose the gene regulation strength within range from 1/40 to 40. The degradation rate of certain gene can be estimated from previous study(Chua et al., 2010). We set the degradation of all genes with same order amplitude as D=0.005/min, the range of which consistent with the available inferred values(Lu et al., 2014) . Genes have different expression or concentration range. To study the gene expression dynamics, it is more convenient to normalize the gene expression as the relative level to be in the range around 1. As shown in Eq. 33, without any gene regulation, X is equal to 1 when A=D. Thus we can represent the gene expression as relative level for each gene and set the basic production rate of the gene or the enzyme as A=0.005. The parameter set of gene regulations are listed in Table S4. The parameters for kinetic velocity equations are from previous studies and listed in Table S5.

## Landscape and Flux Decomposition

For the non-equilibrium biological networks, the dynamics of the whole networks can be described stochastically as:

$$\frac{d\mathbf{x}}{dt} = \mathbf{F}(\mathbf{x}) + \boldsymbol{\zeta} \tag{38}$$

The variable, $\mathbf{x}$ represents the concentrations of genes, enzymes or metabolites. $\mathbf{F}(\mathbf{x})$ is the driving force. The term $\boldsymbol{\zeta}$ represents the noise caused by the fluctuations, of which is statistical nature is assumed as Gaussian and $< \boldsymbol{\zeta}(t)\boldsymbol{\zeta}(t') > = 2\mathbf{D}\delta(t-t')$. $\mathbf{D}$ is the diffusion coefficient tensor describing the level of noise.

The probabilistic evolution in terms of the diffusion equation can be depicted as(NG, 1992):

$$\frac{\partial P}{\partial t} + \nabla \cdot \mathbf{J}(\mathbf{x}, t) = 0 \tag{39}$$

Eq. 39 represents the conservation law of probability. The local change of the probability is equal to the net flux. The probability flux vector $\mathbf{J}$ of the system in space $\mathbf{x}$ is defined as(Feng and Wang, 2011):

$$\mathbf{J}(\mathbf{x}, t) = \mathbf{F}P - \mathbf{D} \cdot \nabla P \tag{40}$$

If the steady state of the system exists, i.e. $\frac{\partial P}{\partial t} = 0$ then $\nabla \cdot \mathbf{J}(\mathbf{x}) = 0$. There are several outcomes.

When $\mathbf{J} = 0$, the zero net flux is the detailed balance condition and the system is in equilibrium. As the definition of flux, $\mathbf{F} = -\mathbf{D} \cdot \nabla U$, where $U = -lnP_{eq}$. So the driving force $\mathbf{F}$ can be represented as a gradient of a potential $U$. The equilibrium probability represents the weight of each state of the equilibrium system, thus the global nature and stability can be quantified as the equilibrium potential(equilibrium probability). The driving force is the gradient of potential, which controls the dynamics of the system.

For the other outcome, $\mathbf{J}(\mathbf{x}) \neq 0$, $\mathbf{J}$ is a rotational curl vector field, due to the divergent free nature of the flux in steady state. For example of $\mathbf{J}(\mathbf{x}) = \nabla \times \mathbf{A}$ in three dimensions, $\mathbf{A}$ is a vector with non-zero curl. $\mathbf{J}(\mathbf{x}) \neq 0$ represents the deviation from detailed balance. Even in steady state, the system is still in non-equilibrium. The magnitude of flux quantifies the degree away from detailed balance or degree away from equilibrium. The probability landscape at steady state represents the weight of each state of the system. It is able to quantify the global nature and stability of the system. Therefore, for non-equilibrium systems and networks, the dynamical driving force F can be decomposed into a gradient of a potential and a curl flow flux(Wang et al., 2008; Feng and Wang, 2011) as:

$$\mathbf{F} = -\mathbf{D}\nabla U + \frac{\mathbf{J_{ss}}(\mathbf{x})}{P_{ss}} \tag{41}$$

The potential U is defined as $U = -lnP_{ss}$ and $P_{ss}$ represents steady state probability distribution. In detailed balance, the underlying dynamics of the system is controlled by the gradient of the potential. The equilibrium potential quantifies the global nature and the stability of the system. For non-equilibrium systems or networks, the global nature and stability are determined by the underlying non-equilibrium potential landscape. The dynamics in this case is determined by both the gradient of potential landscape and probabilistic flux.

## Self Consistent Mean Field Approximation

The evolution of probability distribution on dynamic system can be described by the probabilistic Fokker-Planck diffusion equations in continuous variables such as concentrations. Given the state ($X_1$,$X_2$,...,$X_n$,t), where $X_1$, $X_2$, ...,$X_n$ represent the expression levels of genes or the enzymes, it is difficult to exactly solve high dimensional partial differential equations for the probability P($X_1$,$X_2$,...,$X_n$,t). Here, we apply self consistent mean field approximation for the individual variable. The probability P($X_1$,$X_2$,...,$X_n$,t) can be splitted into the products of the probability of the individual variable, $\prod P(X_i, t)$ according to (Li and Wang, 2013, 2014; Wang et al., 2010a; Sasai and Wolynes, 2003; Zhang and Wolynes, 2014). Thus the dimensionality of the system is reduced to polynomial M × N from exponential $M^N$, which makes the computation and storage tractable. Here M represents the possible number of values of a specific variable X.

It is often difficult to solve self-consistent mean field equation due to its non-linearity. We then start from the moment equations. In principle, once all the moments are known, we can obtain the probability distributions of the dynamic system. Here, Gaussian distribution ansatz is used as an approximation to calculate the probability, and the two moments, mean and variance are needed to compute.

When the diffusion coefficient **D** is small, the moment equations can be approximated to (NG, 1992):

$$\dot{\bar{x}}(t) = \mathbf{F}(\bar{\mathbf{x}(t)}) \tag{42}$$

$$\dot{\boldsymbol{\sigma}}(t) = \boldsymbol{\sigma}(t)\mathbf{A}^{\mathbf{T}}(t) + \mathbf{A}(t)\boldsymbol{\sigma}(t) + 2\mathbf{D}(\bar{\mathbf{x}}(t)) \tag{43}$$

Here, $\bar{x}(t)$ is the mean of the certain variable and $\boldsymbol{\sigma(t)}$ is the covariance matrix of the system evolution. A(t) is a tensor and its matrix element is $\mathbf{A_{ij}} = \frac{\partial F_i(\mathbf{x}(t))}{\partial x_j(t)}$. $\mathbf{A^T}(t)$ is the transpose of $\mathbf{A}(t)$. In terms of these equations, we can solve $\mathbf{x}(t)$ and $\boldsymbol{\sigma}(t)$. The diagonal elements of $\boldsymbol{\sigma}(t)$ is considered. Therefore the evolution of probabilistic distribution for each variable can be expressed by Gaussian approximation determined by the mean and variance:

$$P(x,t) = \frac{1}{\sqrt{2\pi\sigma(t)}} e^{-\frac{(x-\bar{x}(t))^2}{2\sigma(t)}} \tag{44}$$

The equation above represents the expression of the probability for one steady state. For multistable system, the total probability is equal to the combination of the probabilities of all the steady states. The probability of $\mathbf{x}$ in multistable system has the form: $P(\mathbf{x}, t) = \sum w_i P_i(\mathbf{x})$. Here the weight factor $w_i$ for each individual attractors can be obtained through Langevin simulations with multiple initial conditions. In this way, the weight can be computed by the partitions of how many trajectories with different initial conditions fall into different attractors. Finally, the landscape can be quantified through the steady state probability, $U(\mathbf{x}) = -lnP_{ss}(\mathbf{x})$.

## Langevin Stochastic Dynamics Method

For the system in fluctuating environments, the dynamics is often described by the stochastic ordinary differential equations as $\dot{\mathbf{x}} = \mathbf{F}(\mathbf{x}) + \boldsymbol{\zeta}$. Here, $\mathbf{x}(t)$ represents the vector of the gene expression level and enzyme concentration level. $\mathbf{F}(\mathbf{x})$ represents the vector for the driving force through the gene-gene regulations, gene-enzyme regulations/interactions or metabolite-gene regulations/interactions. External fluctuations and intrinsic fluctuations are important to the biology systems(Kærn et al., 2005; Swain et al., 2002). The fluctuation term $\boldsymbol{\zeta}$ is added to the force $\dot{\mathbf{x}} = \mathbf{F}(\mathbf{x})$, the deterministic dynamics of the system. The fluctuation term $\boldsymbol{\zeta}$ is assumed to follow Gaussian distribution and the correlation functions are given as: $\boldsymbol{\zeta_j}(\mathbf{x}, t) >= 0$ and $< \boldsymbol{\zeta_i}(\mathbf{x}, t)\boldsymbol{\zeta_j}(\mathbf{x}, t') >= 2D_{ij}\delta_{ij}\delta(t - t')$ ($\delta_{ij} = 1$ for $i = j$ and $\delta_{ij} = 0$ for $i \neq j$). Here $\delta(t)$ is the Dirac delta function and **D** is diffusion coefficient matrix. The fluctuation term is associated with the intensity of cellular fluctuations either from the environmental external sources or intrinsic sources. Under long time Langevin dynamics simulations, we can obtain the steady state distribution $P(\mathbf{x})$ for the state variable $\mathbf{x}$ through the cumulation of statistics. Finally the potential landscape is obtained by $U = -ln(P(\mathbf{x}))$.

## Path Integral

There are in general many paths from one state to another state. The dominant path is important for biological process and functional switch. Under fluctuations, the dominant path determined based on the path integral method over many possible paths (Wiener, 1921; Onsager and Machlup, 1953).

We can formulate the dynamics for the probability of starting from initial configuration $\mathbf{x_i}$ at t = 0 and end at the final configuration of $\mathbf{x_f}$ at time t. The probability from initial state to the end state is determined by(Wang et al., 2010b, 2011):

$$P(\mathbf{x_f}, t|\mathbf{x_i}, 0) = \int D\mathbf{x}e^{-S(\mathbf{x})} \tag{45}$$

$$S(\mathbf{x}) = \int L[\mathbf{x}(t)]dt \tag{46}$$

In Eq. 45, $S(\mathbf{x})$ is the action, $D\mathbf{x}$ represents the sum of all possible path from state $\mathbf{x_i}$ at t = 0 to the state $\mathbf{x_f}$. L($\mathbf{x}$) is Lagrange and is determined by:

$$L = \frac{1}{4}\mathbf{D}^{-1}\dot{\mathbf{x}} \cdot \mathbf{D}^{-1} \cdot \dot{\mathbf{x}} - \frac{1}{2}\mathbf{D}^{-1}\mathbf{F} \cdot \mathbf{D}^{-1} \cdot \dot{\mathbf{x}} + V \tag{47}$$

$$V = \frac{1}{4}\mathbf{D}^{-1}\mathbf{F} \cdot \mathbf{D}^{-1} + \frac{1}{2}\mathbf{D}\nabla \cdot (\mathbf{D}^{-1} \cdot \mathbf{F}) \tag{48}$$

$\mathbf{D}$ is the diffusion coefficient matrix tensor. The probability of the path is decided by $e^{-S(\mathbf{x})}$. The path is dominant, when the action S is the least.

Not all the paths give the same contribution. We can approximate the path integrals with a set of dominant paths. Because each path is exponentially weighted, contributions of the other sub-leading paths are often small and can be ignored. The optimal paths of the biological paths or transition paths between steady states can be identified.

## Entropy Production Rate

In a nonequilibrium system, exchange in energy and information results the dissipation of energy. It depicts a global physical characterization of the nonequilibrium system. In the steady state, the dissipation of energy is closely associated with the entropy production rate, which could be described as the well-known entropy formula(Qian, 2001):

$$S = -k_{\mathrm{B}} \int P(\mathbf{x}, t)\ln P(\mathbf{x}, t)dx \tag{49}$$

By differentiating the Eq. 49, the increase of the entropy at constant temperature T is shown as follows:

$$T\dot{S} = k_{\mathrm{B}} * T \int (\ln P + 1)\nabla \cdot \mathbf{J}dx = -\int (k_{\mathrm{B}}T\nabla\ln P - \mathbf{F}) \cdot \mathbf{J}dx - \int \mathbf{F} \cdot \mathbf{J}dx = e_p - h_d \tag{50}$$

$$e_p = -\int (k_{\mathrm{B}}T\nabla\ln P - \mathbf{F}) \cdot \mathbf{J}dx \tag{51}$$

$$h_d = \int \mathbf{F} \cdot \mathbf{J} dx \tag{52}$$

As shown in Eq. 50, the entropy can be transformed into the difference between two terms. The former term, $e_p$ is the entropy production rate following Onsager(Onsager and Machlup, 1953), and the latter term, $h_d$ is the mean rate of the heat dissipation. For a steady state, and the entropy production $e_p$ equal to the heat dissipation $h_d$.

## Metabolic Therapeutic Target Prediction

We predict the metabolic therapeutic targets based on the landscape analysis. For each gene or enzyme $x_i$, $F(x_i)$ is changed to $F'(x_i) = F(x_i) + c_i$. The term $c_i$ represents the corresponding changes in activation or inhibition regulations due to the perturbations on the variable. The potential landscape of the four steady state attractors are quantified for the corresponding $c_i$ respectively. If $c_i > 0$, it represents the activation of the gene or the enzyme. If $c_i < 0$, it represents the inhibition of the gene or the enzyme. We define the changes of the cancer OXPHOS state as the degree of therapeutic effect on the OXPHOS cancer type and the changes of the cancer glycolysis state as the degree of therapeutic effect on glycolysis cancer type. If the changes of the barrier height is negative, this leads the instability of certain cancer steady state. This represents the positive effect on the therapeutic target.

We also predicted the effects of combination therapy. This is according to the landscape topography changes in terms of the barrier heights which lead to the higher stability/lower stability for the cancer basins of attraction.

For the malignancy of cancer metabolism oscillation, we aim to weaken the oscillation capability of the limit cycle and drive the system to become mono-stable at normal state by promoting or inhibiting certain genes or enzymes. The oscillation capability can be estimated by the barrier height from the highest point at the center island to the lowest point on the limit cycle.

## References


Brunelle, J. K., Bell, E. L., Quesada, N. M., Vercauteren, K., Tiranti, V., Zeviani, M., Scarpulla, R. C., and Chandel, N. S. (2005). Oxygen sensing requires mitochondrial ROS but not oxidative phosphorylation. *Cell Metabolism*, 1(6):409–414.

Chua, Y. L., Dufour, E., Dassa, E. P., Rustin, P., Jacobs, H. T., Taylor, C. T., and Hagen, T. (2010). Stabilization of hypoxia-inducible factor-1$\alpha$ protein in hypoxia occurs independently of mitochondrial reactive oxygen species production. *Journal of Biological Chemistry*, 285(41):31277–31284.

Courtnay, R., Ngo, D. C., Malik, N., Ververis, K., Tortorella, S. M., and Karagiannis, T. C. (2015). Cancer metabolism and the warburg effect: the role of HIF-1 and PI3k. *Molecular Biology Reports*, 42(4):841–851.

Doe, M. R., Ascano, J. M., Kaur, M., and Cole, M. D. (2011). Myc posttranscriptionally induces HIF1 protein and target gene expression in normal and cancer cells. *Cancer Research*, 72(4):949–957.

Emerling, B. M., Weinberg, F., Snyder, C., Burgess, Z., Mutlu, G. M., Viollet, B., Budinger, G. S., and Chandel, N. S. (2009). Hypoxic activation of AMPK is dependent on mitochondrial ROS but independent of an increase in AMP/ATP ratio. *Free Radical Biology and Medicine*, 46(10):1386–1391.

Faubert, B., Boily, G., Izreig, S., Griss, T., Samborska, B., Dong, Z., Dupuy, F., Chambers, C., Fuerth, B. J., Viollet, B., Mamer, O. A., Avizonis, D., DeBerardinis, R. J., Siegel, P. M., and Jones, R. G. (2013). AMPK is a negative regulator of the warburg effect and suppresses tumor growth in vivo. *Cell Metabolism*, 17(1): 113–124.

Feng, H. and Wang, J. (2011). Potential and flux decomposition for dynamical systems and non-equilibrium thermodynamics: Curvature, gauge field, and generalized fluctuation-dissipation theorem. *The Journal of Chemical Physics*, 135(23):234511.

Hammad, N., Rosas-Lemus, M., Uribe-Carvajal, S., Rigoulet, M., and Devin, A. (2016). The crabtree and warburg effects: Do metabolite-induced regulations participate in their induction? *Biochimica et Biophysica Acta (BBA) - Bioenergetics*, 1857(8):1139–1146.

Harada, H., Itasaka, S., Kizaka-Kondoh, S., Shibuya, K., Morinibu, A., Shinomiya, K., and Hiraoka, M. (2008). The akt/mTOR pathway assures the synthesis of HIF-1$\alpha$ protein in a glucose- and reoxygenation-dependent manner in irradiated tumors. *Journal of Biological Chemistry*, 284(8):5332–5342.

Hasawi, N. A., Alkandari, M. F., and Luqmani, Y. A. (2014). Phosphofructokinase: A mediator of glycolytic flux in cancer progression. *Critical Reviews in Oncology/Hematology*, 92(3):312–321.

Hu, S., Yuan, H., Li, Z., Zhang, J., Wu, J., Chen, Y., Shi, Q., Ren, W., Shao, N., and Ying, X. (2017). Transcriptional response profiles of paired tumor-normal samples offer novel perspectives in pan-cancer analysis. *Oncotarget*, 8(25).

Justus, C., Sanderlin, E., and Yang, L. (2015). Molecular connections between cancer cell metabolism and the tumor microenvironment. *International Journal of Molecular Sciences*, 16(12):11055–11086.

Kærn, M., Elston, T. C., Blake, W. J., and Collins, J. J. (2005). Stochasticity in gene expression: from theories to phenotypes. *Nature Reviews Genetics*, 6(6):451–464.

Landeghem, S. V., Björne, J., Wei, C.-H., Hakala, K., Pyysalo, S., Ananiadou, S., Kao, H.-Y., Lu, Z., Salakoski, T., de Peer, Y. V., and Ginter, F. (2013). Large-scale event extraction from literature with multi-level gene normalization. *PLoS ONE*, 8(4):e55814.

Li, C. and Wang, J. (2013). Quantifying cell fate decisions for differentiation and reprogramming of a human stem cell network: Landscape and biological paths. *PLoS Comput Biol*, 9(8):e1003165.

Li, C. and Wang, J. (2014). Landscape and flux reveal a new global view and physical quantification of mammalian cell cycle. *Proceedings of the National Academy of Sciences*, 111(39):14130–14135.

Li, Y.-N., Xi, M.-M., Guo, Y., Hai, C.-X., Yang, W.-L., and Qin, X.-J. (2014). NADPH oxidase-mitochondria axis-derived ROS mediate arsenite-induced HIF-1$\alpha$ stabilization by inhibiting prolyl hydroxylases activity. *Toxicology Letters*, 224(2):165–174.

Lien, E. C., Lyssiotis, C. A., and Cantley, L. C. (2016). Metabolic reprogramming by the PI3k-akt-mTOR pathway in cancer. In *Metabolism in Cancer*, pages 39–72. Springer International Publishing (2016).

Lim, J.-H., Lee, E.-S., You, H.-J., Lee, J. W., Park, J.-W., and Chun, Y.-S. (2004). Ras-dependent induction of HIF-1$\alpha^{785}$ via the raf/MEK/ERK pathway: a novel mechanism of ras-mediated tumor promotion. *Oncogene*, 23(58):9427–9431.

Lu, M., Jolly, M. K., Onuchic, J., and Ben-Jacob, E. (2014). Toward decoding the principles of cancer metastasis circuits. *Cancer Research*, 74(17):4574–4587.

Marín-Hernández, A., Gallardo-Pérez, J. C., Rodríguez-Enríquez, S., Encalada, R., Moreno-Sánchez, R., and Saavedra, E. (2011). Modeling cancer glycolysis. *Biochimica et Biophysica Acta (BBA) - Bioenergetics*, 1807 (6):755–767.

Marín-Hernández, A., López-Ramírez, S. Y., Mazo-Monsalvo, I. D., Gallardo-Pérez, J. C., Rodríguez-Enríquez, S., Moreno-Sánchez, R., and Saavedra, E. (2014). Modeling cancer glycolysis under hypoglycemia, and the role played by the differential expression of glycolytic isoforms. *FEBS Journal*, 281(15):3325–3345.

Mihaylova, M. M. and Shaw, R. J. (2011). The AMPK signalling pathway coordinates cell growth, autophagy and metabolism. *Nature Cell Biology*, 13(9):1016–1023.

Moreno-Sánchez, R., Saavedra, E., Rodríguez-Enríquez, S., Gallardo-Pérez, J. C., Quezada, H., and Westerhoff, H. V. (2010). Metabolic control analysis indicates a change of strategy in the treatment of cancer. *Mitochondrion*, 10(6):626–639.

Mulukutla, B. C., Khan, S., Lange, A., and Hu, W.-S. (2010). Glucose metabolism in mammalian cell culture: new insights for tweaking vintage pathways. *Trends in Biotechnology*, 28(9):476–484.

NG, V. K. (1992). *Stochastic processes in Chemistry and Physics*. North Holland, 1 edition.

Onsager, L. and Machlup, S. (1953). Fluctuations and irreversible processes. *Physical Review*, 91(6):1505–1512.

Pavlova, N. N. and Thompson, C. B. (2016). The emerging hallmarks of cancer metabolism. *Cell Metabolism*, 23(1):27–47.

Qian, H. (2001). Mesoscopic nonequilibrium thermodynamics of single macromolecules and dynamic entropy-energy compensation. *Physical Review E*, 65(1).

Rodríguez-Enríquez, S., Marín-Hernández, A., Gallardo-Pérez, J. C., and Moreno-Sánchez, R. (2009). Kinetics of transport and phosphorylation of glucose in cancer cells. *Journal of Cellular Physiology*, 221(3):552–559.

Sasai, M. and Wolynes, P. G. (2003). Stochastic gene expression as a many-body problem. *Proceedings of the National Academy of Sciences*, 100(5):2374–2379.

Saunier, E., Benelli, C., and Bortoli, S. (2015). The pyruvate dehydrogenase complex in cancer: An old metabolic gatekeeper regulated by new pathways and pharmacological agents. *International Journal of Cancer*, 138(4): 809–817.

Segel, I. H. (1975). *Enzyme Kinetics*. Wiley, New York.

Song, P. and Zou, M.-H. (2012). Regulation of NAD(p)h oxidases by AMPK in cardiovascular systems. *Free Radical Biology and Medicine*, 52(9):1607–1619.

Swain, P. S., Elowitz, M. B., and Siggia, E. D. (2002). Intrinsic and extrinsic contributions to stochasticity in gene expression. *Proceedings of the National Academy of Sciences*, 99(20):12795–12800.

Trapnell, C., Hendrickson, D. G., Sauvageau, M., Goff, L., Rinn, J. L., and Pachter, L. (2012). Differential analysis of gene regulation at transcript resolution with RNA-seq. *Nature Biotechnology*, 31(1):46–53.

Wang, J., Xu, L., and Wang, E. (2008). Potential landscape and flux framework of nonequilibrium networks: Robustness, dissipation, and coherence of biochemical oscillations. *Proceedings of the National Academy of Sciences*, 105(34):12271–12276.

Wang, J., Li, C., and Wang, E. (2010)a. Potential and flux landscapes quantify the stability and robustness of budding yeast cell cycle network. *Proceedings of the National Academy of Sciences*, 107(18):8195–8200.

Wang, J., Zhang, K., and Wang, E. (2010)b. Kinetic paths, time scale, and underlying landscapes: A path integral framework to study global natures of nonequilibrium systems and networks. *The Journal of Chemical Physics*, 133(12):125103.

Wang, J., Zhang, K., Xu, L., and Wang, E. (2011). Quantifying the waddington landscape and biological paths for development and differentiation. *Proceedings of the National Academy of Sciences*, 108(20):8257–8262.

Wang, S., Zhang, M., Liang, B., Xu, J., Xie, Z., Liu, C., Viollet, B., Yan, D., and Zou, M. H. (2010)c. AMPK 2 deletion causes aberrant expression and activation of NAD(p)h oxidase and consequent endothelial dysfunction in vivo: Role of 26s proteasomes. *Circulation Research*, 106(6):1117–1128.

Wegner, A., Meiser, J., Weindl, D., and Hiller, K. (2015). How metabolites modulate metabolic flux. *Current Opinion in Biotechnology*, 34:16–22.

Wiener, N. (1921). The average of an analytic functional. *Proceedings of the National Academy of Sciences*, 7 (9):253–260.

Wilson, J. E. (2003). Isozymes of mammalian hexokinase: structure, subcellular localization and metabolic function. *Journal of Experimental Biology*, 206(12):2049–2057.

Yuan, G., Khan, S. A., Luo, W., Nanduri, J., Semenza, G. L., and Prabhakar, N. R. (2011). Hypoxia-inducible factor 1 mediates increased expression of NADPH oxidase-2 in response to intermittent hypoxia. *Journal of Cellular Physiology*, 226(11):2925–2933.

Zhang, B. and Wolynes, P. G. (2014). Stem cell differentiation as a many-body problem. *Proceedings of the National Academy of Sciences*, 111(28):10185–10190.

Zhang, M., Dong, Y., Xu, J., Xie, Z., Wu, Y., Song, P., Guzman, M., Wu, J., and Zou, M.-H. (2008). Thromboxane receptor activates the AMP-activated protein kinase in vascular smooth muscle cells via hydrogen peroxide. *Circulation Research*, 102(3):328–337.
